# Single‐Shot Reconfigurable Femtosecond Imaging of Ultrafast Optical Dynamics

**DOI:** 10.1002/advs.202207222

**Published:** 2023-03-04

**Authors:** Peng Wang, Lihong V. Wang

**Affiliations:** ^1^ Caltech Optical Imaging Laboratory Andrew and Peggy Cherng Department of Medical Engineering Department of Electrical Engineering California Institute of Technology 1200 East California Boulevard, Mail Code 138–78 Pasadena CA 91125 USA

**Keywords:** compressed sensing, computational imaging, streak cameras, ultrafast imaging, ultrafast phenomena

## Abstract

Understanding ultrafast dynamics in the femtosecond timescale plays a pivotal role in fundamental research and technology innovation. Spatiotemporal observation of those events in real‐time requires imaging speeds greater than 10^12^ frames per second (fps), far beyond the fundamental speed limits of the ubiquitous semiconductor sensor technologies. In addition, a majority of femtosecond events are non‐repeatable or difficult‐to‐repeat since they either work in a highly unstable nonlinear regime or require extreme or rare conditions to initiate. Therefore, the traditional pump‐probe imaging approach fails since it heavily depends on precise event repetition. Single‐shot ultrafast imaging emerges as the only solution; however, existing techniques cannot reach more than 15×10^12^ fps, and they only record an insufficient number of frames. Compressed ultrafast spectral photography (CUSP) is proposed to overcome these limitations. Here, CUSP's full design space is explored by manipulating the ultrashort optical pulse in the active illumination. Via parameter optimization, an extraordinarily fast frame rate of 219×10^12^ fps is achieved. This implementation of CUSP is also highly flexible, allowing various combinations of imaging speeds and numbers of frames (several hundred up to 1000) to be readily deployed in diverse scientific studies, such as laser‐induced transient birefringence, self‐focusing, and filaments in dielectric media.

## Graphical abstract

Compressed ultrafast spectral photography (CUSP) reaches a new single‐shot imaging speed record of 219 trillion frames per second by parameter optimization. CUSP can be readily reconfigured for different combinations of imaging speeds and numbers of frames. CUSP is implemented for real‐time observation and quantitative characterization of transient birefringence, self‐focusing, filaments in dielectrics, and spatiotemporally chirped complex optical pulses.

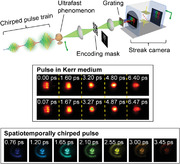

## 1 Introduction

Capturing extremely fast phenomena is instrumental for research in basic physics, biology, chemistry, and technology development. An imaging frame rate beyond a trillion frames per second (Tfps) is required to record and study such phenomena as rogue waves,^[^ ^1^ ^]^ solitons,^[^ ^2^ ^]^ shock waves,^[^ ^3^ ^]^ radiative molecular decay,^[^ ^4^ ^]^ complex optical pulses,^[^ ^5^ ^]^ and chemical reactions.^[^ ^6^ ^]^ However, commercial cameras can image no faster than thousands of frames per second,^[^ ^7^ ^]^ limited by the imaging sensor technologies (e.g., CCD and CMOS). The best high‐speed cameras, based on intensifier gating, can reach only a billion frames per second and acquire at most 30 frames.^[^ ^8^, ^9^ ^]^ An alternative approach, termed the pump‐probe method, has been widely adopted.^[^ ^6^, ^10^, ^11^ ^]^ In this method, the ultrafast event has to be repetitively triggered numerous times while a stroboscopic light with an extremely short time window probes the event at various time delays. This pump‐probe technique fails when the event does not repeat itself precisely or cannot be repeated at all.^[^ ^12^, ^13^ ^]^ Hence, real‐time imaging that works without event repetition has remained a primary demand in ultrafast sciences.

Recent developments in state‐of‐the‐art single‐shot Tfps imaging include frequency‐division imaging,^[^ ^14^ ^]^ light‐in‐flight digital holography,^[^ ^15^ ^]^ non‐collinear optical parametric amplification,^[^ ^16^ ^]^ sequentially‐timed all‐optical mapping,^[^ ^17^ ^]^ frequency‐domain streak imaging,^[^ ^18^ ^]^ and compressed ultrafast photography.^[^ ^19^, ^20^, ^21^ ^]^ Substantial efforts concentrated on achieving greater imaging speeds and larger sequence depths (i.e., the number of frames in each acquisition) have failed to reach beyond 15 Tfps^[^ ^16^ ^]^ and 300 frames,^[^ ^19^, ^20^, ^21^ ^]^ respectively. A promising approach, termed compressed ultrafast spectral photography (CUSP), breaks these barriers and has achieved an unprecedented 70 Tfps imaging speed and nearly 1000‐frame sequence depth simultaneously.^[^ ^22^ ^]^ CUSP synergizes spectral encoding, multi‐pulse illumination, temporal shearing, and compressed sensing. In CUSP, the object is first illuminated by a temporally chirped pulse train so that different wavelengths in each sub‐pulse carry unique time stamps. The transient event is subsequently imaged and spatially encoded by a digital micro‐mirror device (DMD). A dispersive element separates wavelengths in the image plane. Then, a streak camera with a fully opened aperture collects the raw image and distinguishes the sub‐pulses via ultrafast shearing in the direction orthogonal to the wavelength dispersion. Finally, a compressed sensing algorithm extracts a sequence of images from one single acquisition.

In spite of its success in real‐time imaging of femtosecond physical phenomena,^[^ ^22^ ^]^ the previous CUSP system still operated in a sub‐optimal condition, and its design space remained unexplored. Here, in this work, we push the performance of CUSP to its limits by optimizing its parameters, especially the temporal chirp parameter. Rigorous numerical derivation and experimental verification demonstrate the fastest imaging of 219 Tfps with a temporal resolution of 108 fs and a sequence depth of 230 frames. We employed CUSP at this speed to quantitatively study nonlinear light interactions with a dielectric Kerr medium. The easy tunability in the temporal chirp parameter renders CUSP reconfigurable in different combinations of imaging speeds and sequence depths. To showcase such configurability, we switched to an imaging mode of 80 Tfps with 640 frames to observe laser‐induced filamentation in a solid material in real‐time. A slower version of 49 Tfps with 1040 frames was utilized to characterize a spatially complex and spatiotemporally chirped ultrashort optical pulse train.

## 2 Principle of CUSP

**Figure 1:**
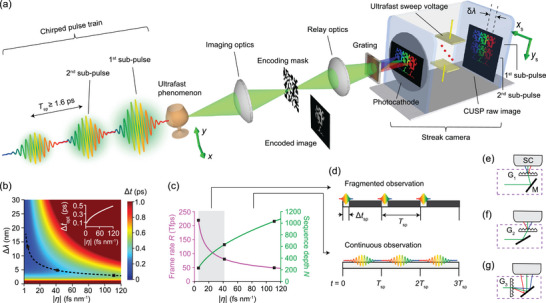
Principle of CUSP. a) Generalized schematic of the CUSP system. An ultrafast phenomenon is probed by a linearly chirped pulse train with a sub‐pulse separation, *T*_sp_, generated from a single femtosecond pulse. A pseudo‐random binary mask is applied at the intermediate image plane, and the encoded image is relayed to the entrance of a streak camera, which distinguishes sub‐pulses by time shearing in the *y*_s_ direction, driven by an ultrafast sweeping voltage. A diffraction grating is inserted between the relay optics and the streak camera to disperse different wavelengths in the *x*_s_ direction. The composition of a raw CUSP image is shown. *δλ* and *δt* stand for the separations between the wavelengths and times of adjacent reconstructed frames, respectively. b) The temporal duration, Δ*t*, of an optical pulse versus the temporal chirp parameter |*η*| and spectral width, Δ*λ*, (equivalent to the spectral resolution in CUSP). The subset shows the minimum temporal width, Δ*t*_opt_, with the optimal spectral resolution, Δ*λ*_opt_, at different temporal chirp conditions. c) Calculated frame rates and sequence depths at different |*η*| values. Gray box represents fragmented observation. d) Illustrations of both fragmented observation (*T*_obs_ < *T*_rec_) and continuous observation ( *T*_obs_ = *T*_rec_ ). *P* is the total number of sub‐pulses used, *T*_rec_ = *PT*_sp_ is the single‐shot recording time, *T*_obs_ = *P*Δ*t*_sp_ is the effective observation time for one acquisition, and Δ*t*_sp_ is the duration of one sub‐pulse. e–g) Implementations of the three configurations of CUSP by using switchable modules consisting of gratings (G_1_–G_3_) and mirrors in front of the streak camera (SC). (e) 219‐Tfps. (f) 80‐Tfps. (g) 49‐Tfps. Black squares in (b) and (c) denote these three configurations.

**Figure** **1**a illustrates how 2D multiplexing of the scene forms a raw CUSP image. To encode time using wavelength, a temporally chirped pulse train is generated by passing a single broadband femtosecond light pulse (70 fs, center wavelength *λ*_0_ = 805 nm) through a pair of high‐reflection plate beamsplitters and a long rod made of highly dispersive glass. Adjusting the distance between the beamsplitter pair changes the sub‐pulse time separation *T*_sp_ [see Equation (S1), Supporting Information]. Rods of different lengths introduce different linear temporal chirp parameters, and thus different time durations for each sub‐pulse [see Equation (S2), Supporting Information]. For spatial encoding, a static pseudo‐random binary pattern, displayed by a DMD, is applied at the intermediate image plane. A diffraction grating inserted between the relay optics and the streak camera splits different wavelengths in the broadband pulse to different positions in the *x*_s_ direction. The encoded dynamic scene is then relayed to the fully opened input aperture of a streak camera. Inside the streak camera,^[^ ^23^ ^]^ a photocathode converts photons to photoelectrons, which are subsequently temporally sheared by an ultrafast sweeping voltage. Temporal shearing effectively separates the sub‐pulses in the *y*_s_ direction. After a phosphor screen converts electrons back to photons, an image intensifier amplifies the signal, and an internal CMOS camera captures the raw CUSP image. While the streak camera captures a time‐sheared spectrum‐dispersed image (*s*‐view), a time‐unsheared spectrum‐undispersed image (*u*‐view) is acquired by incorporating a non‐polarizing beamsplitter and an external camera into the system. Figure S1, Supporting Information, gives the layout of the entire system, and all the optical components are listed in Supporting Information. Figure S2, Supporting Information, explains how the chirped pulse train is generated.

According to the descriptions above, the time stamp of each frame is expressed by *t* (*p*, *λ*) = *pT*_sp_ + *η*(*λ* − *λ*_min_), where *p* is the sub‐pulse index (*p* = 0, 1, 2, 3, 4), *λ* is the wavelength, *η* is the system's temporal chirp parameter (fs nm^−1^), and *λ*_min_ is the minimum wavelength in the effective pulse bandwidth *B*_eff_, defined as the full width at 10% of the peak of the pulse spectrum. The dynamic scene illuminated by this timed pulse train is expressed by optical intensity *I*(*x*, *y*, *t*(*p*, *λ*)). The forward model of the CUSP imaging system has a simplified form of *E* = ***O**I*(*x*, *y*, *t*(*p*, *λ*)), in which *E* includes images from both the *u*‐view (*E*_u_) and *s*‐view (*E*_s_), and ***O*** includes all the operators in the imaging pipeline. Retrieving *I* from the raw images is an under‐sampled ill‐conditioned inverse problem that can be solved by using regularization^[^ ^24^ ^]^ to minimize

(1) $${\mathrm{argmin}}_{I}\left\{\frac{1}{2}∥E-OI{∥}_{2}^{2}+\xi \Phi \left(I\right)\right\}$$

The first term in Equation (1) denotes the discrepancy between the actual (*E*) and the estimated (***O**I*) measurements, and the second term enforces sparsity in the domain defined by the regularizer Φ(*I*). These two terms are balanced by the regularization parameter *ξ*.^[^ ^24^, ^25^, ^26^ ^]^ Regularization allows successful reconstruction only when the dynamic scene satisfies the sparsity requirement in the domain of Φ(*I*).^[^ ^19^, ^24^ ^]^ Specifically, we implemented the two‐step iterative shrinkage/thresholding (TwIST) algorithm for image recovery,^[^ ^22^, ^27^, ^28^ ^]^ and we chose total variation (TV) as Φ(*I*).^[^ ^19^, ^20^, ^22^ ^]^ See Supporting Information for details on the joint operator ***O*** and the reconstruction algorithm.

## 3 Optimization and Configurability of CUSP

The *x*_s_ axis of the internal CMOS camera provides spectral sampling and resolution. *δλ* denotes the wavelength sampling interval, and, correspondingly, *δt* denotes the time interval between adjacent reconstructed CUSP frames [see Equations (S4) and (S5), Supporting Information]. The CUSP system's imaging frame rate is *R* = |*μ*|/(*d*|*η*|)^[^ ^22^ ^]^ [see Equation (S6), Supporting Information], where *μ* is the system's spectral dispersion parameter (µm nm^−1^) and *d* is the streak camera's pixel size (µm). The sequence depth is given by *N* = (*PB*_eff_|*μ*|)/*d* [see Equation (S8), Supporting Information, in Methods], where *P* = 5 is the number of sub‐pulses, and *B*_eff_ is the effective pulse bandwidth used in the illumination (785 to 823 nm). It is straightforward to use a smaller |*η*| and a larger |*μ*| to increase the frame rate. However, this approach does not necessarily offer better temporal resolution. In CUSP, the spectral resolution, Δ*λ*, of a spatial coding unit^[^ ^28^ ^]^ determines the temporal resolution, Δ*t*, in the final reconstructed image. Due to the two competing effects—the time‐bandwidth limit and temporal chirp—there exists an optimal spectral resolution in any chirped pulse that offers the narrowest temporal point‐spread‐function (PSF) for a certain non‐zero |*η*|.^[^ ^17^, ^29^ ^]^ A finer spectral resolution broadens the temporal PSF because the pulse's time‐bandwidth relation is based on the Fourier transformation. A coarser spectral resolution also leads to a broader temporal PSF, in which temporal chirp dominates. See Figure S4, Supporting Information, for definitions of *δλ*, *δt*, Δ*λ*, Δ*t*, *η*, and *B*_eff_; see Supporting Information for the calculation of the optimal condition, and see Figure S5, Supporting Information, for its illustration.

Figure 1b shows the width, Δ*t*, of the numerically calculated temporal PSF versus the spectral resolution Δ*λ* and |*η*|. The black dashed curve traces the valley of Δ*t* (i.e., Δ*t*_opt_), whose values are plotted against |*η*| in the inset. A specific Δ*λ* can be easily realized via adjusting |*μ*|, which is a function of the grating period and the distance between the grating and the streak camera [see Equations (S3) and (S15), Supporting Information]. As shown in Figure 1b, for a smaller |*η*|, in order to reach Δ*t*_opt_, a larger Δ*λ* is required, obtained by a smaller |*μ*| that also reduces sequence depth. We also note that a smaller |*η*| means a shorter Δ*t*_opt_ [see Equation (S10), Supporting Information]. In conclusion, Figure 1c expresses how *R* decreases and *N* increases monotonically versus |*η*| [see Equations (S21) and (S22), Supporting Information]. Figure S6(a), Supporting Information, provides the logarithmic version of these curves. There is a trade‐off between the frame rate and the sequence depth, and their product is a constant, according to Equation (S23), Supporting Information.

To serve diverse applications, by tuning |*η*| using different rod lengths and at the same time selecting the optimal Δ*λ* or |*μ*|, we can reconfigure CUSP with different frame rates and sequence depths. Three example configurations are labeled in Figure 1b,c. The first configuration, at one end of this parameter space, uses a smaller |*η*| to provide a greater *R*. However, an upper bound on *R* must be applied, since the spectrum‐resolving concept of CUSP becomes redundant when fewer than two spectral bands are resolved within the full‐width‐at‐half‐maximum (FWHM) bandwidth, *B*_FWHM_ = 28 nm, of one sub‐pulse [see Equation (S12), Supporting Information]. Therefore, an imaging speed of 219 Tfps turns out to be the fastest achievable by CUSP utilizing our laser's center wavelength and bandwidth. The time interval between neighboring frames is only *δt* = 4.6 fs. The derivation of this new imaging speed limit can be found in Supporting Information. Nevertheless, one caveat of this configuration is that the duration of each sub‐pulse, Δ*t*_sp_, (0.21 ps) is much shorter than the sub‐pulse separation, *T*_sp_. Note that Δ*t*_sp_ is essentially the chirped pulse width with the full bandwidth *B*_eff_ [see Equation (S16) and Figure S6(c), Supporting Information]. According to the experimental results shown in Figure S2(b), Supporting Information, to resolve the neighboring sub‐pulses properly, *T*_sp_ cannot be shorter than 1.6 ps, which is limited by the temporal resolution of CUSP along *y_s_*. When Δ*t*_sp_ < *T*_sp_, black‐out regions exist between sub‐pulses [see Figure 1d], a condition called fragmented observation. As |*η*| increases, Δ*t*_sp_ becomes longer, and when Δ*t*_sp_ ≥ 1.6 ps, there are no black‐out regions between sub‐pulses, which is called continuous observation [see Figure 1d]. In continuous observation, the gap of the beamsplitter pair for pulse‐train generation needs to be finely tuned so that *T*_sp_ = Δ*t*_sp_ , as plotted in Figure S6(c), Supporting Information. The second configuration explored in this work operates at the boundary of these two modes, providing 80‐Tfps imaging. The third configuration provides a sequence depth beyond 1000 frames with a 49‐Tfps imaging speed.

These three configurations are labeled in Figure 1b,c, and Figure S6, Supporting Information; their experimental implementations are illustrated in Figure 1e, Figure S1, and Figure S3, Supporting Information; their relevant parameters are all summarized in Table S1, Supporting Information. The mirrors and gratings before the streak camera are mounted on magnetic bases for easy switching from one configuration to another without disturbing the system's alignment. Figure S2, Supporting Information, shows the dispersion curves, calibrated with a custom‐built monochromator (see Supporting Information and Figure S1, Supporting Information). To show the difference between fragmented observation and continuous observation, we define *T*_obs_ = *P*Δ*t*_sp_ as the total observation time [see Equation (S19), Supporting Information] and *T*_rec_ = *PT*_sp_ as the total recording time [see Equation (S20), Supporting Information]. See Figure S6(d), Supporting Information, for the corresponding plots of *T*_rec_ and *T*_obs_ against |*η*|.

## 4 Results

### 4.1 219‐Tfps Imaging of Light Pulse Propagation in a Fast Kerr Medium

**Figure 2:**
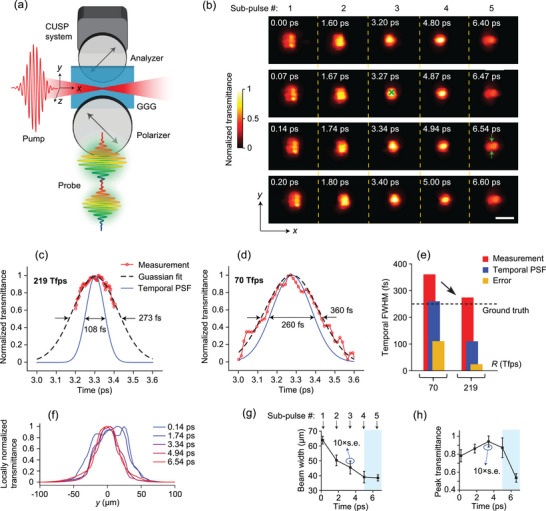
219‐Tfps imaging of light pulse propagation and self‐focusing in a Kerr medium (GGG). (a) Schematic of the experimental setup. A single pump pulse, polarized in the *y* direction, is focused inside the GGG slab from its side. The polarizer and the analyzer have polarization axes aligned at −45° and +45°, respectively, making a typical Kerr gate. b) Example frames of the reconstructed 230‐frame movie (Movie S1, Supporting Information). Transmittance is normalized to the global maximum. The transmittance profile is proportional to the pump's intensity distribution in the *x*‐*y* plane. Each sub‐pulse takes one column, and we collate them since they are spatially separated. Scale bar, 100 µm. c,d) Evolutions of the normalized transmittance at a selected spatial point [green cross in (b)] using (c) 219‐Tfps and (d) 70‐Tfps imaging speeds, respectively. The measurements are fitted in Gaussian functions that are plotted in black dashed lines. The temporal point‐spread‐functions (PSFs) are Gaussian fitted to the experimental calibration data and are plotted in blue solid lines. FWHM, full‐width‐at‐half‐maximum. e) Comparisons between 219‐Tfps and 70‐Tfps CUSP. Red bars represent the FWHMs of CUSP measurements, and blue bars are the FWHMs of temporal PSFs measured experimentally. The black dashed line represents the GGG's response time, our ground truth. Orange bars stand for measurement errors (i.e., the differences between the red bars and black dashed line). f) Transmittance profiles of the pumped Kerr gate along the *y* direction at different times. For clarification, the transmittance is normalized to the local maximum. g,h) Beam width in the vertical direction (g) and peak transmittance (h) over time probed by five consecutive sub‐pulses. The black squares and the error bars represent respectively the averaged values and the standard errors (s.e.) within one sub‐pulse. Since the standard errors are quite small, they are multiplied by 10 to show them clearly. Light cyan boxes indicate the region of self‐focusing.

An ultrashort strong optical field can induce transient birefringence in a dielectric material.^[^ ^30^ ^]^ This ubiquitous light‐matter interaction, termed the Kerr effect, has been widely exploited in mode‐locked lasers,^[^ ^31^ ^]^ optical communications,^[^ ^32^ ^]^ and quantum optics.^[^ ^33^ ^]^ It is also frequently utilized to construct an optical gate (Kerr gate) for ultrafast imaging.^[^ ^34^ ^]^ As our first demonstration, we used 219‐Tfps CUSP to visualize an ultrashort light pulse propagating inside a fast Kerr medium, a gadolinium gallium garnet (Gd_3_Ga_5_O_12_ or GGG)^[^ ^35^ ^]^ crystal slab of 0.5 mm thickness. As illustrated in **Figure** **2**a, transient birefringence was generated by focusing a single femtosecond laser pulse (the pump pulse: 70 fs, 805 nm, and *y*‐polarized) with a peak power density of 2.1×10^12^ W cm^−2^ into the GGG, which functioned as a dynamic wave plate. The instantaneous phase delay, *φ*, between the orthogonal polarization directions *x* and *y* was proportional to the pump pulse intensity. A temporally chirped pulse train (the probe pulse) irradiated the slab in the *z* direction. A pair of linear polarizers, having polarization axes aligned at +45° and −45°, respectively, sandwiched the GGG. As a result, a finite transmittance of (1 − cos *φ*)/2 occurred only where the pump pulse traveled, while everywhere else remained dark, allowing direct imaging of the pump pulse propagation. See Equation (S28), Supporting Information, for the calculation of the Kerr gate's transmittance. Note that for efficient coupling and minimal surface scattering the edge of the GGG slab needs to be polished.

This 219‐Tfps CUSP configuration worked in the fragmented observation regime with Δ*t*_sp_ = 0.21 ps, a fragmented observation time *T*_obs_ = 1.05 ps, and a total recording time *T*_rec_ = 8.00 ps. A sequence depth of 230 frames was compressed into one snapshot when using *P* = 5 sub‐pulses. Figure 2b shows example frames from CUSP reconstruction, and the full sequence is in Movie S1, Supporting Information. The frame indices and sub‐pulse indices are given in addition to the frame time. For simplicity, five snapshots from five sub‐pulses are collated in each row in Figure 2b. As the pump pulse travels and focuses, its growing intensity leads to increased *φ*, therefore transmittance becomes higher. The centroid of the propagating pump pulse matches well with the theoretical estimation based on the GGG's refractive index^[^ ^35^ ^]^ of 1.96.

The ultra‐intense femtosecond pulse drives the electronic charges in the GGG lattice to re‐distribute in accordance with the pulse polarization direction, which imparts anisotropic polarizability, and thus transient birefringence.^[^ ^30^ ^]^ Once the pump is removed, the disturbed charge distribution in the crystal lattice needs a finite, though extremely short, time to relax and return to its original state.^[^ ^35^ ^]^ The normalized transmittance evolution around 3.3 ps [red line in Figure 2c] at a chosen spatial location in Figure 2b (green cross) has a 273‐fs FWHM in the Gaussian fit, suggesting a 250‐fs gating time after deconvolution from the experimentally calibrated temporal PSF with a 108‐fs FWHM [blue solid line in Figure 2c]. This extracted temporal response is faster than that of BGO studied previously.^[^ ^22^, ^35^ ^]^ For a quantitative comparison, we performed the imaging experiment again, using the current state‐of‐the‐art 70‐Tfps CUSP,^[^ ^22^ ^]^ whose experimentally measured temporal PSF, plotted in Figure 2d, has a 260‐fs FWHM. The normalized transmittance and its Gaussian fit in Figure 2d suggest an FWHM of 360 fs. Considering the 250‐fs gating time as our ground truth [black dashed line in Figure 2e], we can obtain the measurement errors by subtracting this ground truth from the FWHMs of the CUSP measurements, as shown in Figure 2e. The 219‐Tfps CUSP is able to reduce the error of the Kerr gating time measurement from 110 fs, for the 70‐Tfps CUSP, to just 23 fs. In other words, our latest and faster CUSP can lower the measurement error by 5 times, a salient advantage. Note that it is challenging to quantify GGG's gating time simply using traditional streak camera imaging with a 1D narrow slit since the streak camera's temporal resolution (> 220 fs) is no shorter than the gating time and much longer than the temporal resolution of CUSP. Also note that the measured gating time of 250 fs is broader than that reported in the previous study^[^ ^35^ ^]^ since a 10 times higher pump pulse fluence is used in our work. This is consistent with the observation and discussion in the literature^[^ ^35^ ^]^ that a higher pump fluence can introduce additional dispersion due to the modulation of the refractive index of the Kerr medium, resulting in a broader gating time.

The intensity‐modulated refractive index is responsible for self‐focusing, a common nonlinear effect.^[^ ^30^ ^]^ The transmittance profiles through the center of the pump beam in the *y* direction at five selected time points are plotted in Figure 2f and their widths are shown in Figure 2g. The widths of the pump pulse, measured in the *y* direction, are plotted in Figure 2f. Both the calculated average width and the standard error (s.e.) of the width probed by each sub‐pulse are shown. It is interesting that the beam width evolution does not show symmetry around its focus, quite unlike linear focusing. The pulse does not diverge after its primary focus that is probed by the third sub‐pulse at around 3.3 ps, but instead further shrinks in width (from 47 to 37 µm) and remains narrow till at least 6.6 ps. We can observe another phenomenon by plotting the peak transmittance [see Figure 2h], which displays asymmetry around the maximum as well. Both the average and the s.e. of the peak transmittance probed by each sub‐pulse are given. A rapid drop in the transmittance after the maximum directly indicates an abnormal decrease in the pump pulse intensity. This occurs because self‐focusing effectively boosts power density by confining photons into a smaller area, and a higher power density can initiate multi‐photon absorption^[^ ^30^, ^36^ ^]^ so that optical energy is lost to absorption and eventually transferred to radiative emission (i.e., fluorescence). The light cyan blocks in Figure 2f,g delimit the self‐focusing regime.

### 4.2 80‐Tfps Imaging of a Laser‐Induced Filament in Glass

**Figure 3:**
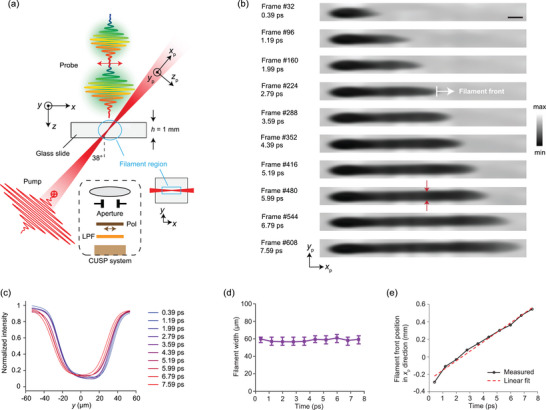
80‐Tfps imaging of a laser‐induced filament in glass. a) Schematic of the experimental setup. A single pump pulse, polarized in the *y* direction, is focused into a glass slide at an oblique angle. *x*_p_‐*y*_p_‐*z*_p_ represents the pump's local coordinates along its propagation. The chirped pulse train probes the filament by imaging its induced opacity in the glass (a shadowgraph). The filament region is bounded in cyan in both the top view and front view (inset). An aperture effectively extends the imaging system's depth‐of‐focus. A polarizer (Pol) and a long‐pass filter (LPF) eliminate unwanted glare and plasma emission, respectively. b) Example frames of the reconstructed 640‐frame movie (Movie S2, Supporting Information). Intensity is normalized to the global maximum. The images are transformed from the *x*‐*y* plane to the pump's local coordinates in the *x*_p_‐*y*_p_ plane. The white arrow represents the propagation direction of the filament front. Scale bars in (b), 50 µm. c) Intensity profiles of the filament along the *y* direction at different times. It is averaged along the *x* direction, and then normalized to the global maximum. d) Filament width over time in ten frames in (b), averaged along the horizontal direction. The error bars indicate the minimum and maximum widths in each frame. e) Position of the filament front in the *x*_p_ direction and its linear fit showing propagation at a constant speed.

Laser‐induced filaments inside dielectric media have been an attractive topic in physics over the past couple of decades.^[^ ^37^ ^]^ Extensive research has investigated the mechanisms of this nonlinear light‐matter interaction,^[^ ^37^, ^38^ ^]^ and filaments have been explored as promising candidates for use in 3D microfabrication^[^ ^39^ ^]^ in photonics and microfluidics. Here, we switched CUSP to the 80‐Tfps imaging mode (at the boundary between fragmented and continuous observations) and recorded the formation and propagation of a filament, which was generated by focusing a single femtosecond pulse (the pump), linearly polarized in the *y* direction, into a clean 1 mm thick glass slide at an oblique angle of 38°. A plano‐convex singlet lens with a 400 mm focal length (effective numerical aperture NA = 0.012) was used for focusing. The chirped pulse train (the probe), polarized in the *x* direction, illuminated the glass slide from the back to create shadowgraphs that directly indicated transient opacity caused by the filament. **Figure** **3**a is a schematic of the experimental setup, in which the filament region is circled in cyan in both the top and front views. Note that a rectangular aperture was applied to the filamentation region in the *x*‐*y* plane to confine the field of view. Since the filament also propagates in the *z* direction, it was necessary to increase the depth of field to image the entire filament from head to tail. This increase was achieved by inserting an aperture at the Fourier plane of the imaging system (see Figure S1, Supporting Information). This solution's side effect of reducing spatial resolution does not intolerably affect the ultimate resolution of the system, since the encoded recording and compressed sensing schemes already compromise the spatial resolution.^[^ ^19^, ^22^, ^28^, ^40^ ^]^ Successful observation of the filament requires suppressing the strong surface glare by using a polarizer whose polarization direction (*x*) is orthogonal to that of the pump. Meanwhile, plasma emission, mostly in the ultraviolet and visible bands, from the surface and interior of the glass is removed by a 715 nm long‐pass spectral filter.

Movie S2, Supporting Information, is a complete CUSP movie containing 640 frames, and ten snapshots are presented in Figure 3b. The filament appears dark because the ultra‐intense electric field in the pump pulse ionizes the atoms to create a plasma cloud of electrons via three‐photon absorption,^[^ ^36^ ^]^ which significantly alters the imaginary part of the permittivity of glass. This alteration results in a larger imaginary part of the refractive index, and thus a distinct reduction in transparency. The pump's peak power density is 1.8×10^14^ W cm^−2^. Since the raw images from CUSP reconstruction are projections in the *x*‐*y* plane, the filament appears shorter than its physical length in the propagation direction. Therefore, we map the raw images to the pump beam coordinate in the *x*_p_‐*y*_p_ plane to obtain the actual filament shape using the pump incident angle of 38° and the glass refractive index of 1.51. At time 7.59 ps, the filament has a measured aspect ratio of ≈20. When the filament propagates, it maintains its average width along the *y* direction, confirmed by the line plots in Figure 3c and the width plot in Figure 3d, where the error bars represent the minimum and maximum widths. This non‐diverging pencil‐like shape is a unique characteristic of filaments that is a consequence of dynamic competitions between focusing and defocusing, caused by optical nonlinearity.^[^ ^30^, ^37^, ^38^ ^]^

Figure 3e shows the position of the filament front in the *x*_p_ direction [marked as a white arrow in Figure 3b] over time and its linear fit measures a propagation speed of *v*_m_ = 0.103 mm ps^−1^. However, this is not the actual propagation speed of filament front since the filament propagates in both *x* and *z* directions, leading to additional time‐of‐flight of the probe light that needs to be compensated. By taking the geometries of both filament and probe beam into account, we can convert *v*_m_ to the actual speed *v*_p_ = 0.194 mm ps^−1^, as detailed in Equations (S29)–(S31) and Figure S8, Supporting Information. Since the speed of light in glass is approximately 0.199 mm ps^−1^, our result proves that the filament front propagates at light speed.

Nonlinear optical transients are susceptible to minute perturbations in the system and thus tend to be unstable and non‐repeatable. Figure S9, Supporting Information, shows multiple pump‐probe shadowgraphs of filaments acquired when the probe pulse is delayed by 8.0 ps relative to the pump pulse. Different from the CUSP imaging experiments in Figure 3, a single femtosecond laser pulse without any chirp was used as the probe light. Consistent with the nature of nonlinearity, the pump pulses of a higher peak power density [1.8×10^14^ W cm^−2^ in Figure S9(a), Supporting Information] generate more unstable and less repeatable filaments than those of a lower peak power density [1.3×10^14^ W cm^−2^ in Figure S9(b), Supporting Information]. Multi‐filamentation also occurs at the tails of the filaments [see #5, #7, #10, and #12 in Figure S9(a), Supporting Information]. Therefore, the pump‐probe method inevitably introduces measurement errors when imaging non‐repeatable events. However, CUSP can avoid such errors by using a single pump pulse without event repetition. Note that similar studies were carried out previously for other transient phenomena such as temporal focusing,^[^ ^20^ ^]^ ultra‐intense light pulse propagation in a Kerr medium,^[^ ^22^ ^]^ and plasma emission in laser‐induced breakdown.^[^ ^13^ ^]^

### 4.3 49‐Tfps Imaging of a Spatiotemporally Complex Pulse Train

**Figure 4:**
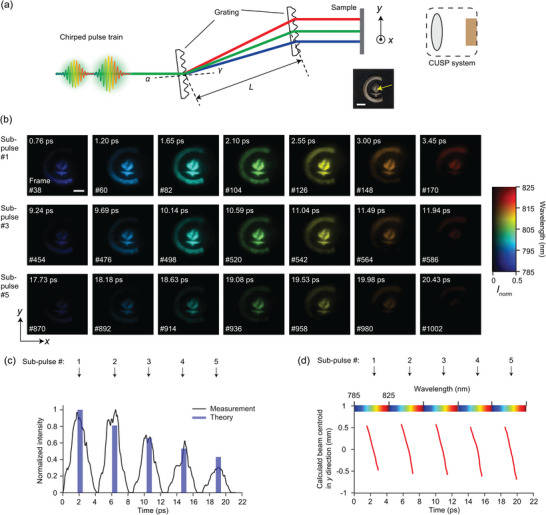
49‐Tfps imaging of a spatiotemporally chirped pulse train. a) Schematic of the experimental setup. Two identical diffraction gratings, distanced by *L*, impart spatial and temporal chirps to the pulse train. Incident angle *α* and diffraction angle *γ* are defined. Inset: a photograph of the printed sample. b) Selected frames from the 1040‐frame movie (Movie S3, Supporting Information). The chirped pulse train scans the sample from bottom to top. The three rows correspond to three sub‐pulses. In the 2D colormap, color represents wavelength and grayscale represents intensity, normalized to the global maximum. Grayscale is saturated at 0.5 to reveal weak intensities. Scale bars in (a) and (b), 1 mm. c) Normalized intensity evolution at one spatial location pointed out by the yellow arrow in (a). The blue bars represent theoretical intensities of the sub‐pulses, estimated by using the beamsplitter pair at 90% reflectivity. d) Calculated centroid of the illumination beam in the *y* direction versus time. The centroid positions were shown when the beam is not cropped by the sample so that the linear spatial chirp is observed.

Spatiotemporally complex pulses have found rapidly growing numbers of applications in optical communications,^[^ ^41^ ^]^ laser physics,^[^ ^42^ ^]^ and optical imaging,^[^ ^43^ ^]^ and real‐time spatiotemporal spectral characterization of such ultrashort pulses plays an essential role in these fields.^[^ ^5^, ^44^ ^]^ In our third experiment, we utilized a grating pair to create a spatiotemporally chirped pulse that irradiated a sample of printed features on a transparency film [see **Figure** **4**a, Equations (S32) and (S33), and Figure S10(a), Supporting Information]. The geometric parameters are given in Supporting Information. This time, we converted CUSP to the 49‐Tfps mode, which can retrieve 1040 frames from one snapshot. Movie S3, Supporting Information, shows the reconstructed data cube, and the example frames are plotted in Figure 4b. Pseudo‐color and grayscale indicate the illumination wavelength and normalized intensity, respectively. Owing to the grating‐pair induced spatial and temporal chirps, each sub‐pulse swiftly sweeps across the sample from bottom to top while the illumination changes from short to long wavelength. Since both chirps are linear, tracing the peak intensity over time in one sub‐pulse is equivalent to analyzing the illuminating pulse spectrum, and the results match well with the direct spectrometer measurement [see Figure S10(b), Supporting Information]. Figure 4c plots the intensity evolution of a chosen spatial pixel [yellow arrow in the inset of Figure 4a]. The five peaks represent five sub‐pulses, and their intensity drops exponentially because of the beamsplitter pair employed in generating the pulse train. The measured intensity matches the theoretical values well. To quantify the spatial chirp of the beam, we computed the beam's centroid in the *y* direction versus time. When the beam is partially cropped by the top and bottom boundaries of the sample, the centroid calculation becomes inaccurate and exhibits nonlinearity over time. Hence, only when the beam is totally inside the sample, we can observe a linear spatial chirp, as plotted in Figure 4d. The measured spatial chirp of 0.09 mm nm^−2^ is close to the theoretical value of 0.10 mm nm^−2^, derived by Equation (S32), Supporting Information.

## 5 Discussion

CUSP is a powerful workhorse for real‐time observation of extremely fast phenomena that are hard to repeat or that occur only once. Compared to all other state‐of‐the‐art single‐shot ultrafast imaging techniques,^[^ ^22^ ^]^ CUSP proves to be superior in both imaging speed and sequence depth. To take full advantage of the potential of CUSP, we explored its design space, especially the temporal chirp parameter, which plays a pivotal role in dictating the overall imaging performances. Our CUSP design accomplished an unprecedented frame rate of 219 Tfps, which is the ultimate speed limit of the current CUSP technology. By imaging and quantifying an ultrashort ultra‐intense light pulse's interaction with a fast Kerr medium in real‐time, we demonstrated a fivefold improvement in the temporal measurement error compared to that of the previous state‐of‐the‐art 70‐Tfps imaging system. As a generic imaging tool, CUSP can be readily tailored for a variety of applications by altering the temporal chirp parameter, *η*, to achieve appropriate combinations of imaging speeds and sequence depths. Thanks to its 2D multiplexing scheme and the principle of compressed sensing, CUSP acquires orders of magnitude more frames in a single snapshot than most alternative methods.

Precise event repetition is indispensable in the traditional pump‐probe imaging approach. CUSP, on the other hand, performs single‐shot recording using a single optical pulse, improving the data acquisition efficiency by orders of magnitude. Most importantly, CUSP can be adapted to image a myriad of interesting phenomena that are inaccessible to the pump‐probe technique, such as chaotic system dynamics,^[^ ^45^ ^]^ sonoluminescence,^[^ ^46^ ^]^ and Cherenkov radiation.^[^ ^47^ ^]^ We have demonstrated the superiority of CUSP over the pump‐probe method when imaging non‐repeatable light‐matter interaction dynamics (see Figure S9, Supporting Information).

Streak camera technology plays a central role in CUSP, challenges, however, exist in operating the streak camera to reach its best performance. The first obstacle is noise in low‐light conditions. We measured the signal‐to‐noise ratios (SNRs) of the streak camera with femtosecond laser illumination of different fluences. An SNR‐limited lower bound in laser fluence is about 0.09 nJ cm^−2^ below which noise becomes too strong to achieve acceptable imaging quality. A second phenomenon, the space‐charge effect, defines the upper bound in laser fluence—5 nJ cm^−2^ above which photoelectrons, generated in the streak tube, reach a high enough density to blur the image due to repulsion among the electrons. As plotted in Figure S11, Supporting Information, it is imperative to ensure that the illumination light stays inside the fluence range defined for decent streak camera imaging.

Oversampling, applied in both spatial and spectral (equivalently temporal) domains, is a widely adopted practice in compressed‐sensing‐based computational imaging techniques, such as compressed ultrafast spectral temporal photography^[^ ^48^ ^]^ and coded aperture snapshot spectral imager,^[^ ^28^, ^40^ ^]^ in which sparsity is enforced in given domains. Thus, it is crucial to decide the optimal sampling rate. To do so, we measured the contrast of the experimentally acquired streak camera images of different sampling rates using the optimal laser fluence. Then, we divided the theoretical spatial resolution (proportional to the sampling rate) by the image contrast, serving as a metric. The metric exhibits a dip at the sampling rate of 16 sensor pixels per DMD encoding unit, corresponding to the 6×6 DMD binning (see Figure S12, Supporting Information). This dip suggests an optimal combination of both high image contrast and high resolution. This analysis explains the difference between the 0.82‐nm spectral sampling and the 13.2‐nm spectral resolution in the 219‐Tfps CUSP, which are translated into the 4.6‐fs frame interval and the 108‐fs temporal resolution.

Degradation in spatial resolution is an inevitable side effect of any single‐shot imaging method enabled by compressed sensing.^[^ ^20^, ^22^, ^28^, ^40^ ^]^ A finite coding size essentially dictates how finely the imaging system can render the scene, and the mixing of multi‐dimensional information scrambles the spatial images. We characterized CUSP's spatial resolution by imaging a spatiotemporally chirped pulse sweeping across a spoke pattern and then analyzing its power spectral density in the spatial frequency domain. Based on the experimental analyses shown in Figure S13, Supporting Information, the spatial resolution can be 3× worse than that in static image acquisition. However, one promising avenue to overcoming this resolution loss is to add more views with distinct operators.^[^ ^13^, ^21^ ^]^ In addition to spatial resolution, CUSP's temporal resolution was also characterized using the same experiment, as shown in Figure S13, Supporting Information. The FWHM of the intensity evolution at a selected spatial location in the reconstructed movie was defined as the temporal resolution. Note that the same temporal PSF was used in the first experimental demonstration shown in Figure 2c. Furthermore, the success of CUSP reconstruction hinges on the sparsity of images in certain domains, which is satisfied in our demonstrations.

Of note, the maximum imaging speed of 219 Tfps demonstrated here applies to the CUSP system using our femtosecond laser with an 805 nm center wavelength and a 28 nm bandwidth. The generalized speed limit and temporal resolution limit are derived in Supporting Information, and the corresponding plots are in Figure S7, Supporting Information. Note that the imaging speed limit is proportional to the laser's FWHM bandwidth and inversely proportional to the square of the center wavelength. Using a commercially available femtosecond laser with *λ*_0_ = 800 nm and *B*_FWHM_ = 50 nm, CUSP can theoretically reach an imaging speed of 420 Tfps, with a temporal resolution of 55 fs and a sequence depth of 230 frames. Such analysis again endorses CUSP as the fastest real‐time imaging modality with the greatest number of frames in a single acquisition.

On the software side, prospective research directions include optimizing the regularizer,^[^ ^24^ ^]^ developing application‐oriented encoding patterns,^[^ ^49^ ^]^ and using image reconstruction based on machine learning.^[^ ^50^ ^]^ On the hardware side, the time‐bandwidth limit, a version of the uncertainty principle, fundamentally bounds the temporal resolution.^[^ ^29^ ^]^ Additionally, although a streak camera is vital in CUSP, its disadvantages include intrinsic jitter, the space‐charge effect, limited field of view, poor spatial resolution, low quantum efficiency, and high cost. Thus, replacing the streak camera with alternative shearing mechanisms^[^ ^51^ ^]^ could lead to novel systems with even better performance.

## Conflict of Interest

The authors disclose the following patent applications: WO2016085571 A3 (L.V.W.), U.S. Provisional 62/298,552 (L.V.W.), and U.S. Provisional 62/904,442 (L.V.W. and P.W.).

## Author Contributions

P.W. conceived the system design, built the system, performed the experiments, developed the reconstruction algorithm, analyzed the data, and wrote the manuscript. L.V.W. supervised the project. All authors revised the manuscript.

## Supporting information

Supporting Information

Supporting Movie 1

Supporting Movie 2

Supporting Movie 3

## Data Availability

The data that support the findings of this study are available from the corresponding author upon reasonable request.
