# Supplementary material for: Single‐Shot Reconfigurable Femtosecond Imaging of Ultrafast Optical Dynamics
Source: Adv Sci (Weinh). 2023 Mar 4;10(13):2207222. doi: 10.1002/advs.202207222 (PMC10161092; doi:10.1002/advs.202207222)
Supplement: Supplementary file 1 — Supporting Information [file ADVS-10-2207222-s002.pdf]

ADVANCED SCIENCE
Open Access

Supporting Information

for *Adv. Sci.*, DOI 10.1002/advs.202207222

Single-Shot Reconfigurable Femtosecond Imaging of Ultrafast Optical Dynamics

*Peng Wang and Lihong V. Wang**

Supplementary Information

Single-shot reconfigurable femtosecond imaging of ultrafast optical dynamics

Peng Wang[1] and Lihong V. Wang[1,*]

[1] Caltech Optical Imaging Laboratory, Andrew and Peggy Cherng Department of Medical Engineering, Department of Electrical Engineering, California Institute of Technology, 1200 East California Boulevard, Mail Code 138-78, Pasadena, CA 91125, USA

*Corresponding author: LVW@caltech.edu

1. CUSP system details

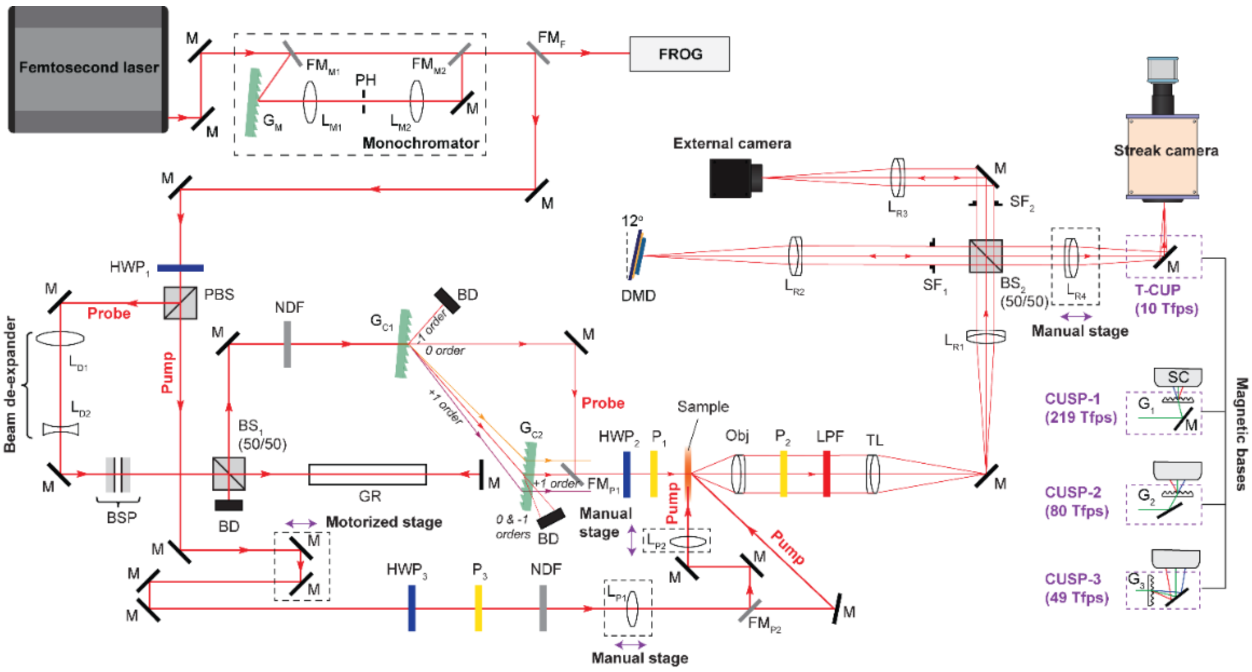

Fig. S1. Detailed schematic of the CUSP system. BD, beam dump; BS, 50/50 (R/T) non-polarizing cube beamsplitter; BSP, beamsplitter pair; DMD, digital micro-mirror device; FM, flip mirror; FROG, Frequency-resolved optical gating; G, diffraction grating; GR, glass rod; HWP, half-wave plate; L, lens; LPF, long-pass filter; M, mirror; NDF, neutral density filter; Obj, objective lens; P, polarizer; PBS, polarizing beamsplitter; PH, pinhole; TL, tube lens; SC, streak camera; SF, spatial filter. Detailed information and their usages are described below.

Equipment

The entire system is schematically shown in Fig. S1. A femtosecond laser (Coherent, Libra HE) is the light source. The laser's pulse width is 70 fs, and its spectrum is centered at 805 nm, with an FWHM bandwidth of 28 nm. It has a repetition rate of 1000 Hz and a maximum pulse energy of 4 mJ. The original $1/e^2$ beam diameter is 15 mm. The custom-built monochromator uses a 1200 lp/mm reflective diffraction grating (Thorlabs, GR25-1208), a 100 mm lens (Thorlabs, LA1050), another 100 mm lens (Thorlabs, LA1509), and a pinhole (Thorlabs, P50C). A half-wave plate (Ealing, 45-7929-000) and a polarizing beamsplitter (Thorlabs, PBS25-780-HP) separate the pump and probe beams. The pump beam path includes a half-wave plate (Thorlabs, AHWP05M-980), a linear polarizer (Thorlabs, GL10-B), a 400 mm lens (Thorlabs, LA1725), and a 25 mm lens (Thorlabs, LJ1075L2). The optical delay line is mounted on a motorized

translation stage (Physik Instrumente, PLS-85). The probe beam path includes a 150 mm lens (Thorlabs, AC254-150-B), a negative 50 mm lens (Thorlabs, LC1715), a pair of 90/10 plate beamsplitters (Thorlabs, BSX11R), a 50/50 non-polarizing beamsplitter (Thorlabs, BS014), SF11 glass rods of different lengths (Newlight Photonics, SF11G1050-AR800, SF11G1300-AR800, SF11G1400-AR800), a pair of 600 lp/mm transmissive diffraction gratings (Thorlabs, GT25-06V), a half-wave plate (Thorlabs, WPH10M-780), and a linear polarizer (Thorlabs, LPVIS100-MP2). The imaging system includes a linear polarizer (Thorlabs, LPNIRE100-B), a long-pass filter (Thorlabs, FGL715), a 50/50 non-polarizing beamsplitter (Thorlabs, BS014), a DMD (Texas Instruments, LightCrafter 3000), an external CCD camera (Point Grey, GS3-U3-28S4M), 150 mm lenses (Thorlabs, AC254-150-B), and a streak camera (Hamamatsu, C6138). Two irises (Thorlabs, ID25) are used as spatial filters at the Fourier planes of the imaging system. The 219-Tfps imaging of the Kerr effect used a 4× objective lens (Thorlabs, RMS4X) and a 100 mm lens (Thorlabs, AC254-100-B). The 80-Tfps imaging of a filament used a 10× objective lens (Thorlabs, RMS4X) and a 75 mm lens (Thorlabs, AC254-075-B). The 49-Tfps imaging of spatiotemporally chirped pulses used a 75 mm lens (Thorlabs, AC254-075-B) and a 25 mm lens (Thorlabs, AC127-025-B). A 300 lp/mm transmissive diffraction grating (Thorlabs, GTI25-03) was used in both 219-Tfps and 49-Tfps imaging, and a 600 lp/mm transmissive diffraction grating (Thorlabs, GT25-06V) was used in 80-Tfps imaging. All the mirrors are ultrafast mirrors with high reflectivity and low group delay dispersion (Thorlabs, UM10-AG and UM10-45A).

System characterization

FM_{M1} and FM_{M2} are used to switch to the monochromator module for dispersion calibration. The monochromator comprises a reflective diffraction grating, G_M; two lenses, L_{M1} and L_{M2}; and a 50 µm pinhole, PH. FM_F is used to switch to the FROG module for characterization of the original femtosecond pulse.

Pump beam path

PBS splits the original pulse into the pump and probe beams, and HWP_1 is used to adjust their intensity ratio. In the pump beam, an optical delay line finely adjusts the time delay between the pump and probe for accurate detection of ultrafast phenomena. HWP_3 and P_3 control the pump's polarization state. Two lenses, L_{P1} and L_{P2}, are used for the first demo, Kerr effect imaging, while only L_{P1} is used for the second demo, filament imaging. Both lenses are mounted on translation stages for focus adjustment. FM_{P2} switches between the first and second experiments.

Probe beam path

In the probe beam, L_{D1} and L_{D2} make a telescope to contract the probe beam to one-third of its original diameter. The BSP contains two 90/10 (R/T) plate beamsplitters facing each other to create a pulse train. In the pulse train, the time delay between the neighboring sub-pulses is determined by the physical gap, h_{b}, between the plate beamsplitters, expressed by

$$T_{\mathrm{sp}} = \frac{2h_{\mathrm{b}}}{c}. \tag{S1}$$

Here, c is the speed of light. One of the beamsplitters is on a linear translation stage to finely control this time delay.

The pulse is stretched by GR. BSP and GR together generate the temporally chirped pulse train for active illumination. The amount of temporal chirp depends on the length, g, of the pulse-stretching rod made of high-dispersion glass SF11. Usually, we define η as

$$\eta = \bar{\eta} g, \tag{S2}$$

in which $\bar{\eta}$, in units of fs/nm/mm, is the intrinsic temporal chirp parameter of the glass. Since rod lengths have to be in discrete values and we had only a handful of options, we designed the three CUSP configurations carefully so that the available rod lengths gave $|\eta|$ as close to the target values as possible (see Table S1). We are aware that a grating pair with a folding mirror can also serve as a pulse stretcher with the capability of continuous tuning of pulse duration. However, we did not use that option due to its low optical throughput and complexity.

For system compactness, BS_1 enables a double pass through GR. The diffraction grating pair G_{C1} and G_{C2} generates spatiotemporally chirped pulses by using +1 diffraction order, which is exploited in the third demonstration experiment using 49-Tfps imaging. The 0 diffraction order without dispersion works as the probe light for the first and second experiments, using 219-Tfps and 80-Tfps imaging, respectively. HWP_2 and P_1 control the probe's polarization state. FM_{P1} switches between the no-spatial-chirp illumination (first and second demos) and spatial-chirp illumination (third demo).

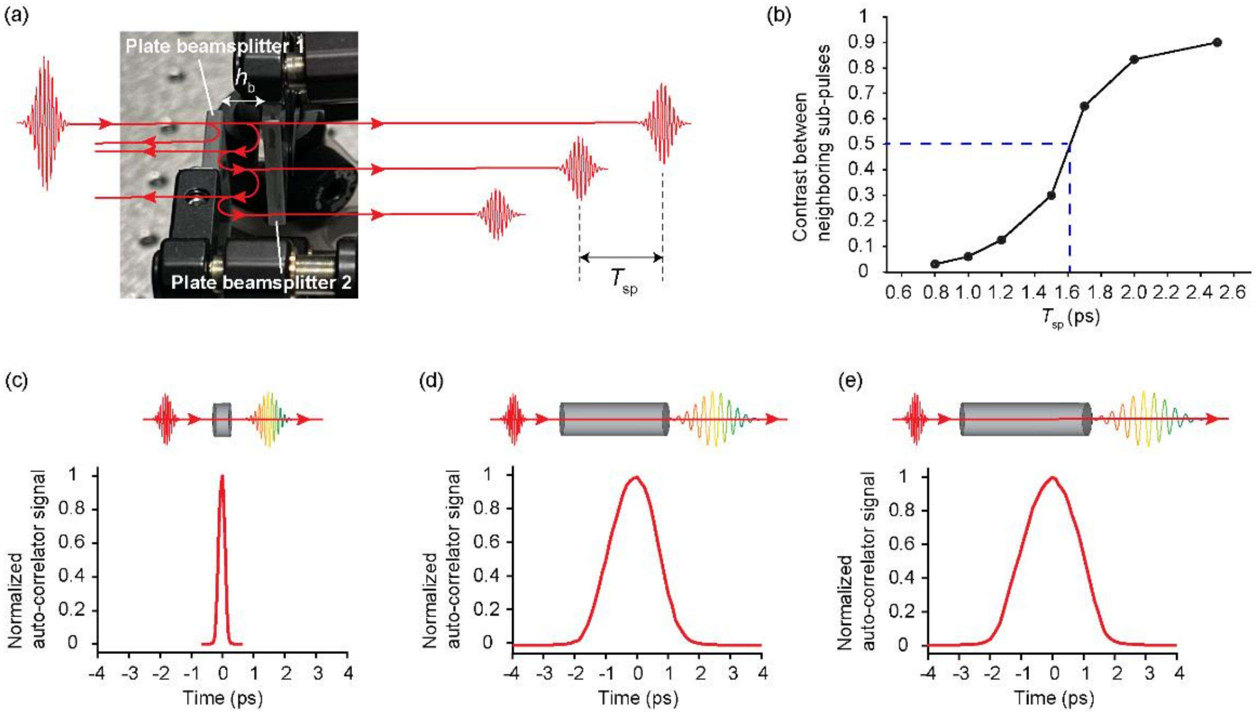

Fig. S2. Generation of a chirped pulse train. (**a**) Photograph of the setup that converts a single femtosecond light pulse to a pulse train by bouncing back and forth multiple times inside an off-resonance cavity consisting of two parallel plate beamsplitters. The separation between neighboring sub-pulses T_{sp} is determined by the gap h_{b}. (**b**) Intensity contrast between neighboring sub-pulses in CUSP reconstruction when the sub-pulse separation T_{sp} is varied by translating one beamsplitter in the beamsplitter pair. A simple pulse train without spatial or temporal chirps illuminates a spoke pattern. We employ the 80-Tfps mode in this experimental test. As marked by the blue dashed lines, we choose 1.6 ps as the minimum T_{sp} value, defined by a contrast threshold of 0.5 when the peaks of neighboring sub-pulses are distinguishable. (**c**)-(**e**) A femtosecond light pulse is chirped and stretched by highly dispersive glass rods of different lengths: (**c**) 10 mm, (**d**) 75 mm, (**e**) 90 mm. The chirped pulses are measured by a custom-built intensity auto-correlator.

Imaging system

Different combinations of objective lenses, Obj, and tube lenses, TL, give the different magnification factors used in the three experiments. Polarizer P_2 allows certain polarization state to be transmitted for detection and removes unwanted polarization. LPF blocks plasma emission. L_{R1}, L_{R2}, L_{R3}, and L_{R4} have the same focal lengths for 1:1 image relays. L_{R4} is mounted on a translation stage to compensate for small path length discrepancies in different CUSP configurations. Different CUSP modules (diffraction gratings and

mirrors), along with the previous T-CUP module (mirror only), are mounted on individual magnetic bases for easy swapping between different imaging modes.

Dispersion calibration for three CUSP configurations

Based on the diffraction grating's geometry, the dispersion parameter of the CUSP system is

$$|\mu| = \frac{l}{\Lambda \cos \varphi}. \tag{S3}$$

In Equation (S3), l is the distance between the grating and the streak camera's entrance, Λ is the grating period, and φ is the diffraction angle.

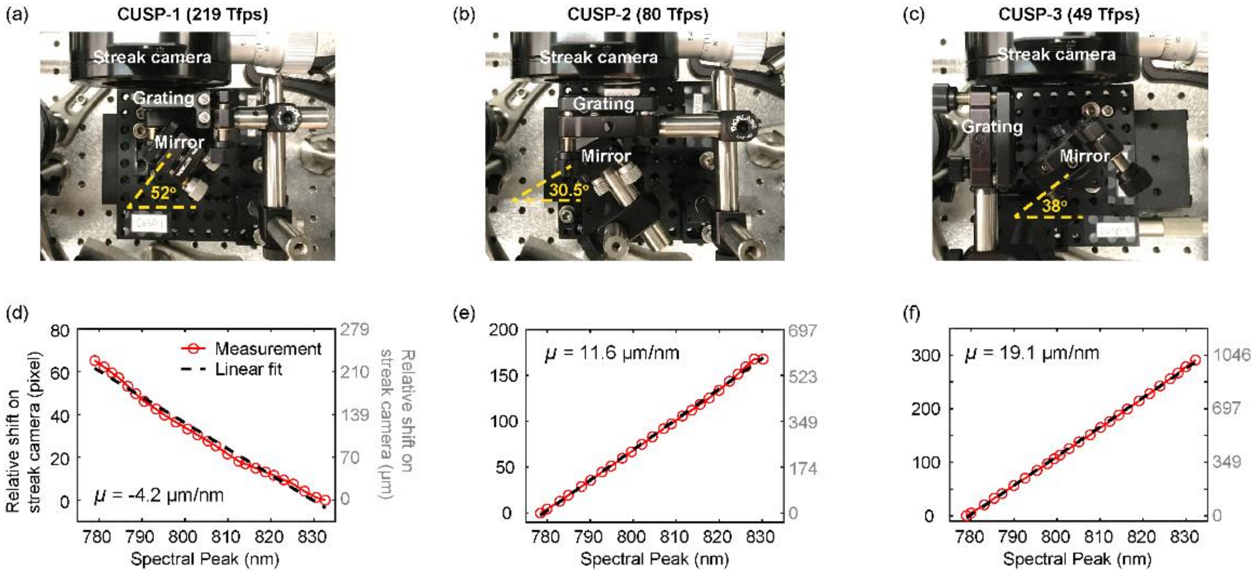

Fig. S3. Three CUSP configurations and their dispersion calibrations. (**a**)-(**c**) Photographs of the optical setups in front of the streak camera, configured for (**a**) 219-Tfps, (**b**) 80-Tfps, and (**c**) 49-Tfps. The tilt angles of the mirrors are labeled in yellow. (**d**)-(**f**) Dispersion calibration curves, measured using the custom-built monochromator (red circles). Black dashed lines are linear fits. The left y-axis is the relative shift on the streak camera in units of sensor pixels, and the right y-axis is in units of micrometers, calculated from the sensor pixel size of 6.45 µm and the streak camera's internal magnification of 1.85×.

Digital micro-mirror device

In the DMD, each micro-mirror (also known as a DMD pixel) is a flat metallic reflector that can be individually addressed to turn to either +12° (ON) or –12° (OFF). We tilt the DMD chip by 12° and collect

the encoded scene in a retro-reflection mode (Fig. S1)[1-3]. Here, we opt to use 6×6 binning in DMD pixels to form one DMD code. Since each DMD pixel is 9.45 μm in size, each individual DMD code is 56.7 μm × 56.7 μm, which can be resolved by the relay optics with 0.08 NA. When the binning size is too small, individual DMD code becomes indistinguishable due to random noise and the space-charge effect present in the streak camera, degrading CUSP reconstructions. On the other hand, when the binning size gets too large, CUSP resolves poorly in both space and time, since the spatial and temporal resolutions are proportional to the binning size[1,4,5]. Based on characterization experiments, this 6×6 binning is optimal (see Fig. S12 for more details). According to the streak camera's pixel size of 6.45 μm and internal magnification of 1.85×, each spatial pixel occupies 3.49 μm at the streak camera's entrance. Therefore, we need to use a total of 16×16 streak camera pixels to cover one DMD code. We define 16 as the spatial sampling rate, S, of our system (see Fig. S12 for more details).

Streak camera

In the streak camera[6] with fully opened aperture, first, a photocathode converts photons to photoelectrons, which are accelerated via a high-voltage anode. An ultrafast linear voltage ramp temporally shears these electrons in the vertical y_s direction. The highest sweeping speed is 100 fs per pixel (time range 50 ps), or equivalently 10 THz. A microchannel plate amplifies the signals by generating secondary electrons. Finally, a phosphor screen converts the electrons back to photons, and an internal CCD camera captures a single image. Note that we take the mode-locking signal from the femtosecond laser to trigger a delay generator (Stanford Research, DG645), and then the delayed signal triggers the streak camera for image acquisition. An accurate delay is required to compensate for the time difference between the electronic and optical signals.

2. CUSP performance calculation

Frame rate and sequence depth.

Based on the definition of the dispersion parameter $|\mu|$, we can express the wavelength difference between neighboring pixels by

$$\delta\lambda = \frac{d}{|\mu|}. \tag{S4}$$

Here, d is the streak camera's sensor pixel size. Based on the definition of the linear temporal chirp parameter, the time difference between the neighboring pixels is

$$\delta t = |\eta|\delta\lambda = \frac{d|\eta|}{|\mu|}, \tag{S5}$$

which defines the time interval between neighboring CUSP frames. Since the imaging frame rate is the inverse of δt, we have

$$R = \frac{1}{\delta t} = \frac{|\mu|}{d|\eta|}. \tag{S6}$$

In addition, the number of wavelengths sampled in one sub-pulse with the full effective bandwidth B_{eff} is

$$N_{\mathrm{sp}} = \frac{B_{\mathrm{eff}}}{\delta\lambda} = \frac{B_{\mathrm{eff}}|\mu|}{d}. \tag{S7}$$

This number is equivalent to the number of pixels that the dispersed spectrum occupies, and it also corresponds to the number of frames contained in one sub-pulse. Therefore, when using P sub-pulses, the sequence depth is

$$N = PN_{\mathrm{sp}} = \frac{PB_{\mathrm{eff}}|\mu|}{d}. \tag{S8}$$

See the definitions of all the variables used in the spectral and time domains in Fig. S4.

Optimal performance.

The time duration, Δt, of any Fourier-transform-limited light pulse with a temporal chirp depends on its spectral width, $\Delta\lambda$, as follows:[7,8]

$$\Delta t = \sqrt{\left(\frac{2{\lambda_0}^2 \ln 2}{\pi c \Delta\lambda}\right)^2 + (\eta\Delta\lambda)^2}\,, \tag{S9}$$

where λ_0 is the center wavelength of the pulse, c is the speed of light, and η is the temporal chirp parameter. The first term under the square root represents the time-bandwidth relation based on Fourier transformation, and the second term represents temporal chirp. A typical plot of Equation (S9) is in Fig. S5(a), and a 2D plot at different $|\eta|$ values is in Fig. 1(b). The minimum temporal spread

$$\Delta t_{\mathrm{opt}} = 2\lambda_0 \sqrt{\frac{\ln 2\,|\eta|}{\pi c}}, \qquad \text{when } |\eta| \geq |\eta|_{\min}, \tag{S10}$$

occurs at the optimal spectral width

$$\Delta \lambda_{\mathrm{opt}} = \lambda_0 \sqrt{\frac{2\ln 2}{\pi c |\eta|}}, \qquad \text{when } |\eta| \geq |\eta|_{\min}. \tag{S11}$$

Note that Equations (S10) and (S11) are valid only when $|\eta| \geq |\eta|_{\min}$. Here, $|\eta|_{\min}$ is defined as when there are two resolvable spectra within the full-width-at-half-maximum (FWHM) B_{FWHM} of the original pulse spectrum. The number of resolvable spectral bands in one sub-pulse is defined by $N_{\mathrm{res}} = B_{\mathrm{FWHM}}/\Delta\lambda_{\mathrm{opt}}$. Then, when $N_{\mathrm{res}} = 2$, $|\eta|_{\min}$ becomes

$$|\eta|_{\min} = \frac{8\ln 2}{\pi c}\left(\frac{\lambda_0}{B_{\mathrm{FWHM}}}\right)^2. \tag{S12}$$

See the plot of N_{res} with the marked position of $|\eta|_{\min}$ in Fig. S6(b). The 219-Tfps imaging configuration is selected when N_{res} approaches 2, defining the upper bound on the CUSP's imaging speed.

When $N_{\mathrm{res}} < 2$, or equivalently $|\eta| < |\eta|_{\min}$, the CUSP concept of time encoding in wavelength becomes redundant and the CUSP system should be replaced by the previously described T-CUP system[2], the imaging speed of which is substantially compromised (maximum of 10 Tfps[2]). Therefore, it is unnecessary to define $\Delta\lambda_{\mathrm{opt}}$ and Δt_{opt} when $|\eta| < |\eta|_{\min}$.

Based on Equation (S2), we can choose the correct length of the stretching rod to obtain the desired temporal chirp parameter. Once $|\eta|$ is fixed, we need to make sure that the imaging system can resolve $\Delta\lambda_{\mathrm{opt}}$. The spectral resolution of the CUSP system is defined as

$$\Delta\lambda_{\mathrm{sys}} = \max(\Delta\lambda_{\mathrm{grat}}, \Delta\lambda_{\mathrm{CS}}). \tag{S13}$$

Typically, the spectral resolution based on grating dispersion, denoted by $\Delta\lambda_{\mathrm{grat}}$, takes into account the number of illuminated grating periods and the sensor pixel size. Calculations show that the spectral resolution of our hardware implementation is far beyond what is needed to resolve $\Delta\lambda_{\mathrm{opt}}$.

Hence, we need to match the spectral resolution $\Delta\lambda_{\mathrm{CS}}$ enabled by the compressed sensing scheme with the desired $\Delta\lambda_{\mathrm{opt}}$. In other words, $\Delta\lambda_{\mathrm{opt}} = \Delta\lambda_{\mathrm{sys}} = \Delta\lambda_{\mathrm{CS}}$. In CUSP, as in other coded imaging

techniques[4,5], $\Delta\lambda_{\mathrm{CS}}$ is primarily determined by the DMD's coding size on the streak camera's entrance, meaning that we need to design the system so that one DMD code spatially occupies one resolvable spectral band, based on linear dispersion by a grating. Applying our optimal spatial sampling rate $S = 16$ (i.e., the number of streak camera pixels per dimension within one coding unit) and considering Equation (S11), we can derive the wavelength difference between neighboring camera pixels using

$$\delta\lambda = \frac{\Delta\lambda_{\mathrm{CS}}}{S} = \frac{\Delta\lambda_{\mathrm{opt}}}{S} = \frac{\lambda_0}{S}\sqrt{\frac{2\ln 2}{\pi c|\eta|}}, \qquad \text{when } |\eta| \geq |\eta|_{\mathrm{min}}. \tag{S14}$$

Considering Equation (S4), we can obtain the required dispersion of the system:

$$|\mu| = \frac{d}{\delta\lambda} = \frac{Sd}{\Delta\lambda_{\mathrm{opt}}} = \frac{Sd}{\lambda_0}\sqrt{\frac{\pi c|\eta|}{2\ln 2}}, \qquad \text{when } |\eta| \geq |\eta|_{\mathrm{min}}. \tag{S15}$$

Equation (S15) suggests what dispersion parameter $|\mu|$ is required to achieve optimal performance when the temporal chirp parameter $|\eta|$ is given. Based on Equation (S3), the proper grating period [G_1–G_3 in Figs. 1(e)-1(g) and Fig. S1] and the distance between the grating and streak camera are chosen to meet the required dispersion parameter.

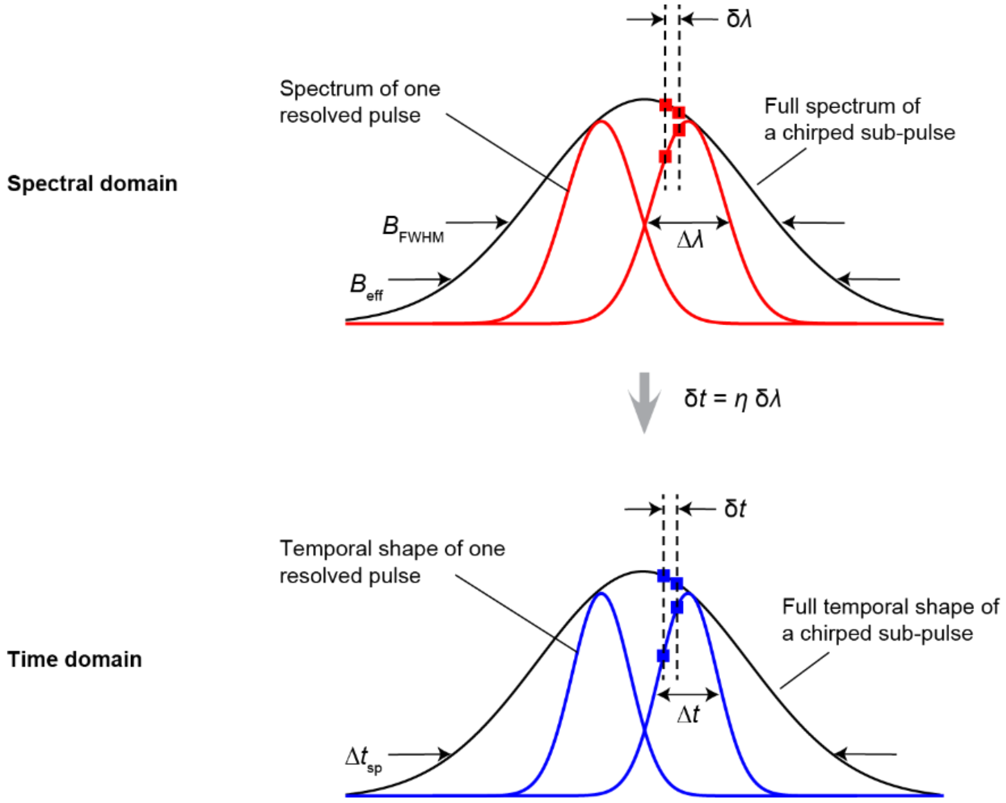

Fig. S4. Definitions of the notations in the spectral and time domains. Here, $\Delta\lambda$ and Δt denote respectively the bandwidth and the duration of a resolved pulse in the final CUSP image. $\delta\lambda$ denotes the wavelength sampling interval along x_s of the internal CMOS camera, and correspondingly δt denotes the time interval between adjacent reconstructed CUSP frames [see Equations (S4) and (S5)]. The temporal chirp parameter $|\eta|$ links $\delta\lambda$ and δt, whereas $\Delta\lambda$ and Δt are related by Equation (S9). B_{FWHM} and B_{eff} represent the full-width-at-half-maximum and the effective full bandwidth of the spectrum of one sub-pulse, respectively. Δt_{sp} represents the sub-pulse duration corresponding to B_{eff}.

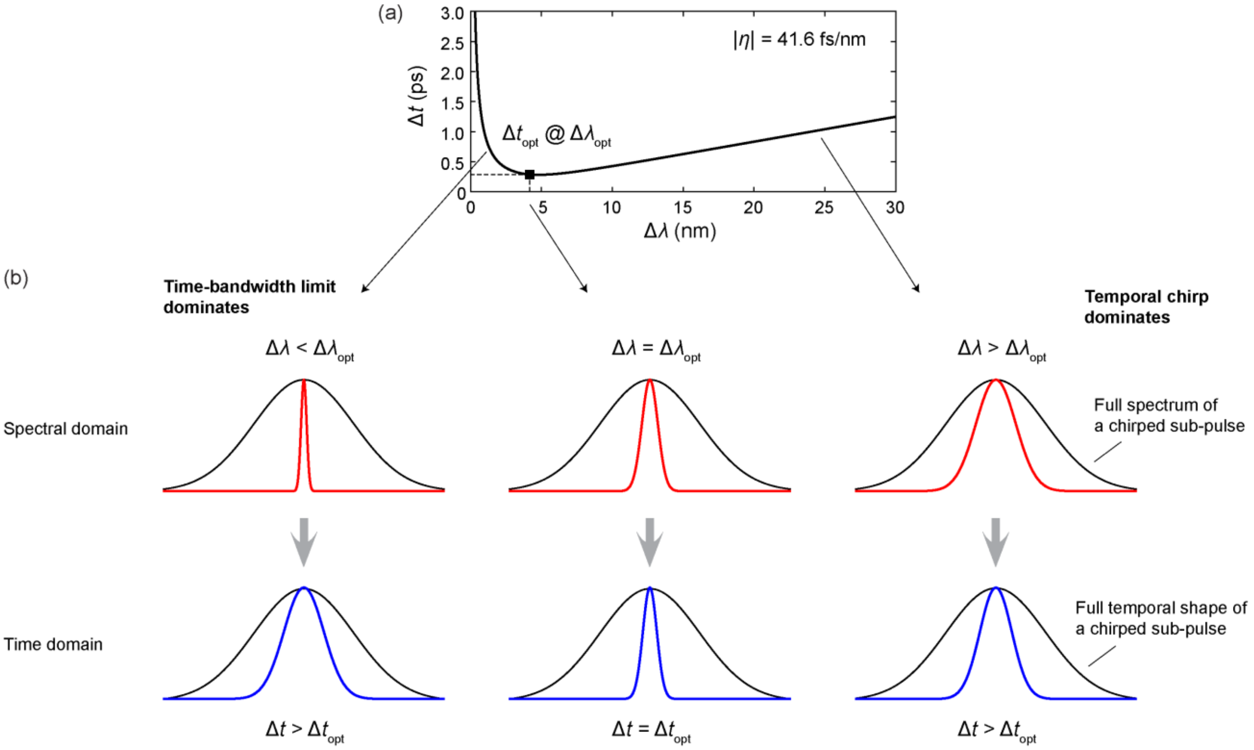

Fig. S5. Optimal spectral resolution for CUSP operation. (**a**) Temporal duration Δt of a resolved pulse with the temporal chirp parameter $|\eta| = 41.6\ \mathrm{fs/nm}$ and different spectrum widths $\Delta\lambda$, representing different spectral resolutions. Δt is calculated using Equation (S9). A minimum Δt_{opt} is reached at the optimal spectrum resolution, $\Delta\lambda_{\mathrm{opt}}$. See Equations (S10) and (S11). (**b**) Illustration of the relationship between $\Delta\lambda$ and Δt in a temporally chirped pulse. Black curves represent the spectrum and temporal shape of the original chirped full pulse, red curves represent the resolved spectra of different $\Delta\lambda$, and blue curves represent the resolved temporal pulses. In both situations—time-bandwidth-limit dominance ($\Delta\lambda < \Delta\lambda_{\mathrm{opt}}$) and temporal-chirp dominance ($\Delta\lambda > \Delta\lambda_{\mathrm{opt}}$), the resolved pulse is longer than the optimal situation ($\Delta t > \Delta t_{\mathrm{opt}}$).

Total observation time and total recording time.

When $|\eta|$ becomes large, the sub-pulse duration, Δt_{sp}, can be approximated by

$$\Delta t_{\text{sp}} = |\eta| B_{\text{eff}}, \qquad \text{when } |\eta| \geq |\eta|_{\text{min}}. \tag{S16}$$

Here, B_{eff} represents the effective full bandwidth of the pulse, as defined in the main text. The sub-pulse separation can be expressed by the following piecewise equation:

$$T_{\text{sp}} = \begin{cases} T_{\text{sp}}{}^{\text{min}}, & \text{when } |\eta|_{\text{min}} \leq |\eta| < |\eta|_{\text{bd}} \\ |\eta| B_{\text{eff}}, & \text{when } |\eta| \geq |\eta|_{\text{bd}} \end{cases}. \tag{S17}$$

The boundary between fragmented and continuous observations is

$$|\eta|_{\text{bd}} = \frac{T_{\text{sp}}{}^{\text{min}}}{B_{\text{eff}}}. \tag{S18}$$

In Equations (S17) and (S18), the minimum sub-pulse separation is $T_{\text{sp}}{}^{\text{min}} = 1.6$ ps, as analyzed in Fig. S2(b), therefore, $|\eta|_{\text{bd}} = 41.6\ \text{fs/nm}$. This boundary $|\eta|_{\text{bd}}$ is labeled in the plots of Δt_{sp} and T_{sp} in Fig. S6(c).

Therefore, the total observation time, T_{obs}, and the total recording time, T_{rec}, are calculated by

$$T_{\text{obs}} = P\Delta t_{\text{sp}} = P|\eta| B_{\text{eff}}, \qquad \text{when } |\eta| \geq |\eta|_{\text{min}}. \tag{S19}$$

$$T_{\text{rec}} = PT_{\text{sp}} = \begin{cases} PT_{\text{sp}}{}^{\text{min}}, & \text{when } |\eta|_{\text{min}} \leq |\eta| < |\eta|_{\text{bd}} \\ P|\eta| B_{\text{eff}}, & \text{when } |\eta| \geq |\eta|_{\text{bd}} \end{cases}. \tag{S20}$$

Both T_{obs} and T_{rec} are plotted against $|\eta|$ in Fig. S6(d).

Trade-off of frame rate and sequence depth.

Taking Equation (S15) into Equation (S6), we can also express the frame rate by

$$R = \frac{1}{\delta t} = \frac{S}{|\eta|\Delta\lambda_{\text{opt}}} = \frac{S}{\lambda_0}\sqrt{\frac{\pi c}{2\ln 2\,|\eta|}}, \qquad \text{when } |\eta| \geq |\eta|_{\text{min}}. \tag{S21}$$

Similarly, taking Equation (S15) into Equation (S8), we can also express the total sequence depth by

$$N = P\frac{B}{\Delta\lambda_{\text{opt}}/S} = \frac{PBS}{\lambda_0}\sqrt{\frac{\pi c|\eta|}{2\ln 2}}, \qquad \text{when } |\eta| \geq |\eta|_{\text{min}}. \tag{S22}$$

Eventually, by multiplying Equations (S21) and (S22), we find that the product of the imaging speed and sequence depth (termed RNP) is a constant:

$$\mathrm{RNP} = RN = \frac{\pi c P B S^2}{2 \ln 2 \, {\lambda_0}^2}, \tag{S23}$$

independent of η. Equation (S23) holds in both the fragmented and continuous observation regimes.

Based on Equations (S21) and (S22), we can achieve both faster R and more N by increasing the sampling rate, S, while $|\eta|$ is intact, but this compromises spatial resolution, as aforementioned. Therefore, the optimal value of $S = 16$ (i.e., 6×6 DMD binning) is selected to balance both R (also N) and spatial resolution.

<u>Imaging speed limit of a generalized system.</u>

The maximum imaging speed of 219 Tfps is achieved using a femtosecond laser with λ_0 of 805 nm and B_{FWHM} of 28 nm. To derive the generalized equation of imaging speed limit, we need to first let the number of resolvable spectra $N_{\mathrm{res}} = 2$. Then taking Equation (S12) into Equation (S21), we can express the maximum imaging speed by

$$R^{\mathrm{best}} = \frac{\pi c}{4 \ln 2} \frac{S B_{\mathrm{FWHM}}}{{\lambda_0}^2}. \tag{S24}$$

We can also take Equation (S12) into Equation (S10) to get the best temporal resolution:

$$\Delta {t_{\mathrm{opt}}}^{\mathrm{best}} = \frac{4\sqrt{2} \ln 2}{\pi c} \frac{{\lambda_0}^2}{B_{\mathrm{FWHM}}}. \tag{S25}$$

The highest imaging speed, R^{best}, and the best temporal resolution, $\Delta {t_{\mathrm{opt}}}^{\mathrm{best}}$, are functions of the laser's center wavelength, λ_0, and bandwidth, B_{FWHM}, plotted in Fig. S7.

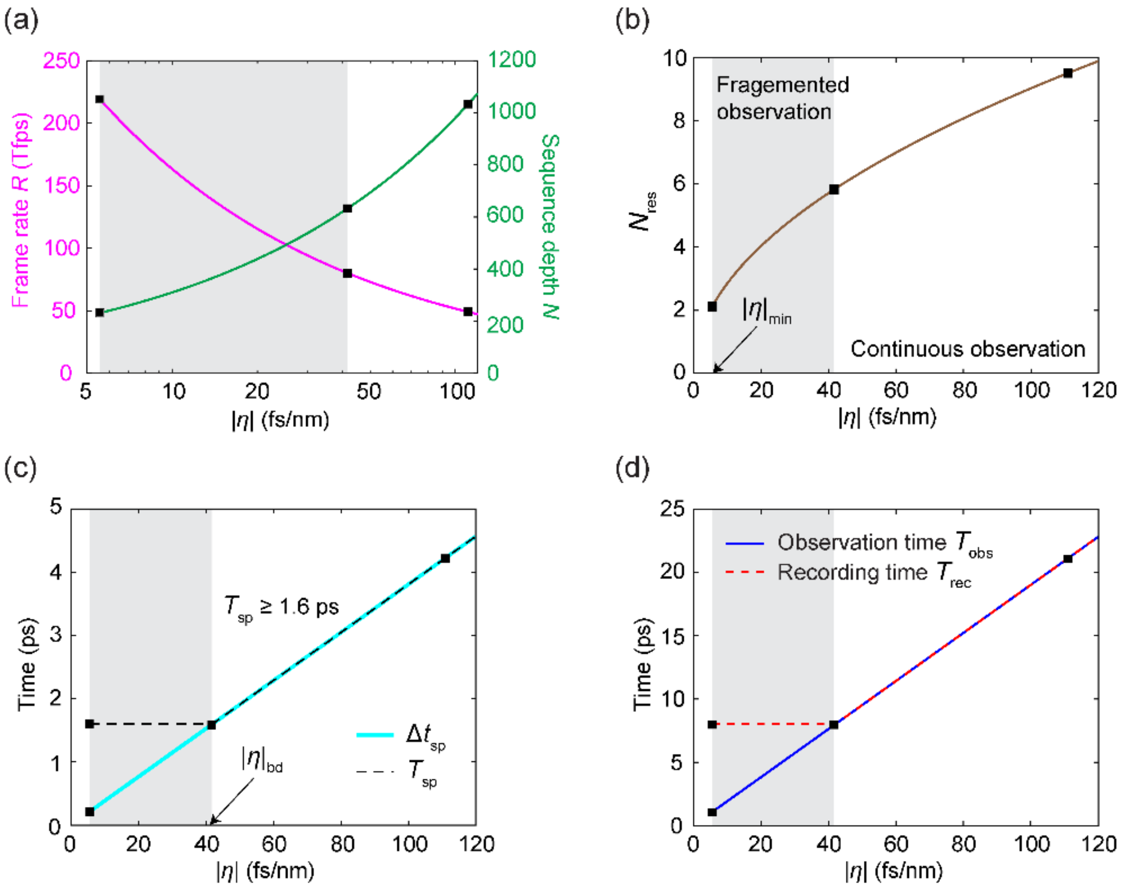

Fig. S6. Additional performance plots. (**a**) Frame rate, R, and sequence depth, N, versus the temporal chirp parameter, plotted in logarithmic scale, as calculated by Equations (S21) and (S22), respectively. (**b**) Number of resolvable spectra, N_{res}, within the full-width-at-half-maximum of the original pulse spectrum, B_{FWHM}. The minimum temporal chirp parameter, $|\eta|_{\min}$, when $N_{\mathrm{res}} = 2$, calculated by Equation (S12), is marked. (**c**) Durations of a sub-pulse, Δt_{sp}, and sub-pulse separation, T_{sp}, versus $|\eta|$, as calculated by Equations (S16) and (S17), respectively. T_{sp} is kept at 1.6 ps for fragmented observation and $T_{\mathrm{sp}} = \Delta t_{\mathrm{sp}} \geq$ 1.6 ps for continuous observation. The boundary between fragmented observation and continuous observation at $|\eta|_{\mathrm{bd}}$ when $\Delta t_{\mathrm{sp}} = 1.6$ ps, as calculated by Equation (S18), is marked. (**d**) The total effective observation time, T_{obs}, and the total recording time, T_{rec}, versus $|\eta|$, as calculated by Equations (S19) and (S20), respectively. The black squares represent the three example configurations. The gray boxes represent fragmented observation.

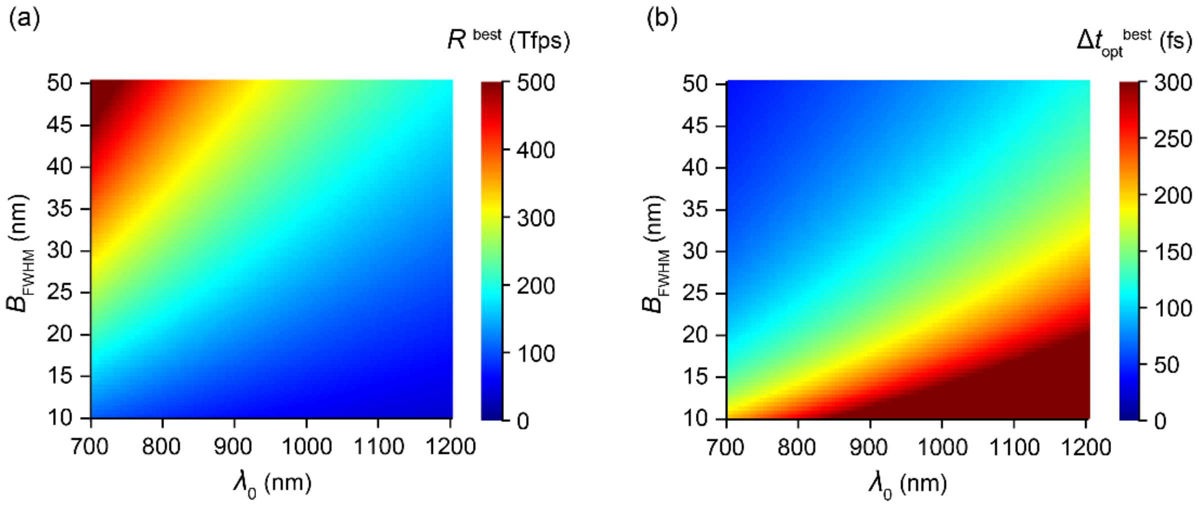

Fig. S7. The best imaging speeds and temporal resolutions of CUSP when using femtosecond lasers with different parameters. The center wavelengths range from 700 nm to 1200 nm, and the bandwidths are between 10 nm and 50 nm, representing typical specifications in commercial laser products. (**a**) The best imaging speed, R^{best}, is calculated by Equation (S24). (**b**) The best temporal resolution, $\Delta t_{\text{opt}}{}^{\text{best}}$, is calculated by Equation (S25).

3. Image acquisition and reconstruction

The complete forward imaging model of CUSP is given by

$$\begin{bmatrix} E_{\mathrm{u}} \\ E_{\mathrm{s}} \end{bmatrix} = \begin{bmatrix} \boldsymbol{T}\boldsymbol{Q}_{\mathbf{u}}\boldsymbol{F}_{\mathbf{u}} \\ \alpha\boldsymbol{T}\boldsymbol{S}_{\boldsymbol{t}}\boldsymbol{Q}_{\mathbf{s}}\boldsymbol{S}_{\boldsymbol{\lambda}}\boldsymbol{D}\boldsymbol{F}_{\mathbf{s}}\boldsymbol{C} \end{bmatrix} I\big(x, y, t(p, \lambda)\big)\,, \tag{S26}$$

where $\boldsymbol{C}$ represents spatial encoding; $\boldsymbol{F}_{\mathbf{u}}$ and $\boldsymbol{F}_{\mathbf{s}}$ describe spatial low-pass filtering due to the optics in the u-view and s-view, respectively; $\boldsymbol{D}$ represents image distortion in the s-view with respect to the u-view; $\boldsymbol{S}_{\boldsymbol{\lambda}}$ denotes spectral dispersion in the x_{s} direction; $\boldsymbol{Q}_{\mathbf{u}}$ and $\boldsymbol{Q}_{\mathbf{s}}$ are the quantum efficiencies of the external CCD and the streak camera, respectively; $\boldsymbol{S}_{\boldsymbol{t}}$ denotes temporal shearing in the y_{s} direction; $\boldsymbol{T}$ represents spatio-spectro-temporal integration; and α is the calibrated energy ratio between the streak camera and the external CCD. All these operators together compose our joint operator, $\boldsymbol{O}$.

For CUSP reconstruction, total variation was introduced as the regularizer in Equation (1) in main text, defined by

$$\begin{aligned} \Phi(I) = &\sum_{p}\sum_{\lambda}\sum_{x,y}\sqrt{\begin{matrix}\big[I\big(x+\delta x, y, t(p,\lambda)\big) - I\big(x, y, t(p,\lambda)\big)\big]^2 + \\ \big[I\big(x, y+\delta y, t(p,\lambda)\big) - I\big(x, y, t(p,\lambda)\big)\big]^2\end{matrix}} \\ &+\sum_{x}\sum_{y}\sum_{p,\lambda}\sqrt{\begin{matrix}\big[I\big(x, y, t(p+1,\lambda)\big) - I\big(x, y, t(p,\lambda)\big)\big]^2 + \\ \big[I\big(x, y, t(p,\lambda+\delta\lambda)\big) - I\big(x, y, t(p,\lambda)\big)\big]^2\end{matrix}}\,, \end{aligned} \tag{S27}$$

in which δx, δy, and $\delta\lambda$ are the sampling step sizes in the x, y, and λ dimensions, respectively.

To implement CUSP reconstruction, it is necessary to accurately measure the joint operator $\boldsymbol{O}$, especially the encoding matrix. Its calibration procedure was described in previous publications[3,9]. In the experiments of Kerr and filament imaging, the reconstruction outputs are the transmittance multiplied by both the illumination spectrum [see Fig. S10(b)] and the relative sub-pulse intensities [see Fig. 4(c)]. Therefore, numerical compensation for the spectrum shape and sub-pulse intensities is required in post-processing to reach the final results shown in Fig. 2, Fig. 3, Move S1, and Movie S2. Note that the parallel plate beamsplitter pair (see Fig. S1) can generate an infinite number of sub-pulses; however, the sub-pulse intensity drops exponentially, decreasing signal-to-noise ratios. The later sub-pulses can introduce crosstalk to the first 5 sub-pulses. Therefore, in image post-processing, we initially reconstructed the first 7 sub-pulses and then selected frames from the first 5 sub-pulses as our results to eliminate the effect of crosstalk.

4. Additional descriptions in the imaged ultrafast optical dynamics

Transmittance and response time of the Kerr gate

The intensity-dependent transmittance of the Kerr gate[10] is

$$T_{\mathrm{Kerr}} = \frac{1-\cos(k_{\mathrm{GGG}}\Delta n l_{\mathrm{Kerr}})}{2} = \frac{1-\cos\left(k_{\mathrm{GGG}}\kappa_{\mathrm{GGG}} I_{\mathrm{pump}} l_{\mathrm{Kerr}}\right)}{2}. \tag{S28}$$

In Equation (S28), k_{GGG} is the angular wavenumber in GGG, Δn is the pump-induced birefringence, κ_{GGG} is GGG's nonlinearity coefficient, I_{pump} is the pump pulse intensity, and l_{Kerr} is the interaction length between the pump pulse and the probe pulse. When $I_{\mathrm{pump}} = 0$, the Kerr gate has the minimum transmittance $T_{\mathrm{Kerr}} = 0$. In our previous work[3], we studied the instability of T_{Kerr} in response to the random fluctuations of I_{pump}, which shows the incapability of the pump-probe method and demonstrates the necessity of single-shot imaging.

The 250-fs response time of GGG was derived by deconvolution. The Kerr temporal response profile of GGG is approximated by a Gaussian function. We first convolved CUSP's temporal PSF (see Fig. S13f) with the Kerr response profiles of different FWHMs (from 100 fs to 500 fs with a 5-fs step size). The FWHMs of the convolved curves were calculated and compared to the experimentally measured width of 273 fs. The Gaussian function that gives the best match to this width was selected as the true Kerr response profile.

Calculation of filament propagation speed

In order to convert the measured speed in Fig. 3(e) to the actual speed of filament propagation, we need to resort to the schematic shown in Fig. S8. First, at time t_1 the probe light is at depth z_2 and the filament front is at depth z_1. The probe light meets the filament front at time $t_2{}'$ and then propagates to depth z_1 at time t_2, which is the time recorded by CUSP. The probe light propagates at a speed of c/n, in which c is the speed of light in vacuum and n is the refractive index of glass. The filament forms along the direction of pump pulse at an angle θ and propagates at a speed of v_{p}. It has propagated a distance of x_{p} before it meets the probe light. Therefore, based on simple geometry, we have

$$\frac{c}{n}(t_2 - t_1) = \frac{c}{n}(t_2{}' - t_1) + x_{\mathrm{p}}\cos\theta\,. \tag{S29}$$

Then, we can have

$$t_2{}' = t_2 - \frac{x_{\mathrm{p}}\cos\theta}{c/n}\,. \tag{S30}$$

Since $x_{\mathrm{p}} = v_{\mathrm{p}}(t_2' - t_1)$, we can express the propagation speed of filament front

$$v_{\mathrm{p}} = \frac{x_{\mathrm{p}}}{t_2' - t_1} = \frac{x_{\mathrm{p}}}{t_2 - \frac{x_{\mathrm{p}} \cos\theta}{c/n} - t_1} = \frac{1}{\frac{t_2 - t_1}{x_{\mathrm{p}}} - \frac{\cos\theta}{c/n}} = \frac{1}{\frac{1}{v_{\mathrm{m}}} - \frac{\cos\theta}{c/n}}. \tag{S31}$$

In Equation (S31), $v_{\mathrm{m}} = x_{\mathrm{p}}/(t_2 - t_1)$ stands for the propagation speed directly measured from the CUSP results shown in Fig. 3(e). Thus, using $v_{\mathrm{m}} = 0.103\,\mathrm{mm/ps}$, $\theta = 24°$ and $n = 1.51$ in glass, we can obtain the actual propagation speed of filament to be $v_{\mathrm{p}} = 0.194\,\mathrm{mm/ps}$, which is consistent with the speed of light in glass.

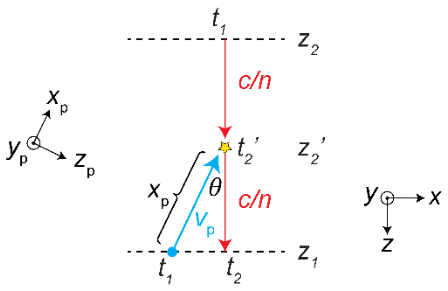

Fig. S8. Schematic explaining the calculation of filament propagation speed. The object space coordinate system (x-y-z) and the filament's local coordinate system (x_{p}-y_{p}-z_{p}) are both defined as in Fig. 3(a). The red arrows represent the direction of probe light propagation, and the cyan arrow represents the direction of filament front propagation.

Instability of laser-induced filament

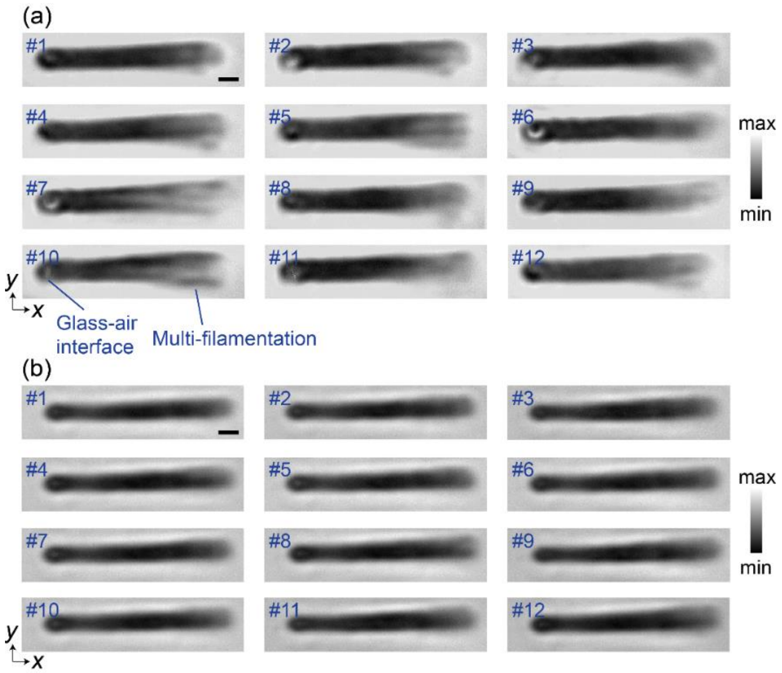

Fig. S9. Two groups of snapshots of filaments captured by the pump-probe approach. They are shadowgraphs in the x-y plane and the grayscale colormap represents the transmitted probe light intensity. The numbers represent the indices of consecutive pump pulses. The probe light is an unchirped femtosecond pulse, arriving 8.0 ps later than the pump pulse. (**a**) The pump pulses have a peak power density of 1.8×10^{14} W/cm^2, same with that used in the 80-Tfps CUSP imaging experiment shown in Fig. 3 and Movie S2. (**b**) The pump pulses have a peak power density of 1.3×10^{14} W/cm^2. Scale bars: 50 μm.

<u>Spatial and temporal chirps induced by a grating pair.</u>

The spatial chirp, $\varepsilon_{\mathrm{gp}}$, and temporal chirp, η_{gp}, induced by a grating pair can be estimated by Equations (S32) and (S33), respectively. The incident angle, α, diffraction angle, γ, and grating distance, L, are defined in Fig. 4(a).

$$\varepsilon_{\mathrm{gp}} = \frac{L\cos\alpha}{\Lambda_{\mathrm{gp}}(\cos\gamma)^2}, \tag{S32}$$

$$\eta_{\mathrm{gp}} = \frac{L\lambda_0}{c\Lambda_{\mathrm{gp}}^2(\cos\gamma)^2}. \tag{S33}$$

In order to achieve the temporal chirp of $\eta_{\mathrm{gp}} = 161.1\ \mathrm{fs/nm}$ and the spatial chirp of $\varepsilon_{\mathrm{gp}} = 0.10\ \mathrm{mm/nm}$, we need to use $\alpha = 6.7°$, $\gamma = 21.3°$, $L = 145.6\ \mathrm{mm}$, and $\Lambda_{\mathrm{gp}} = 600\ \mathrm{lp/mm}$. Combining η_{gp} and the

−50.1 fs/nm temporal chirp induced by a glass rod, we assign the overall temporal chirp of the system to be 111.0 fs/nm, leading to the 49 Tfps imaging speed (see Table S1).

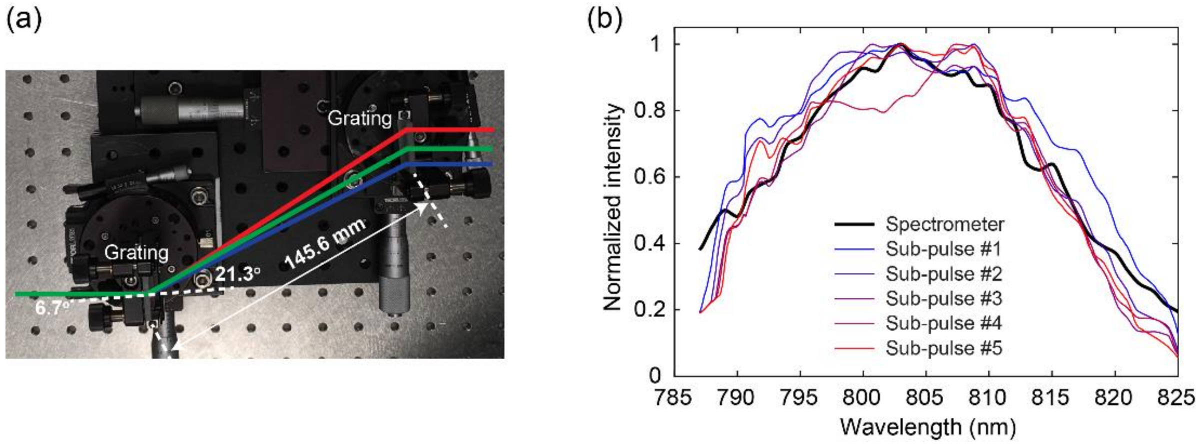

Fig. S10. Additional analysis of the spatiotemporally complex pulse train. (**a**) Photograph of the optical module of a grating pair to introduce spatial and temporal chirps simultaneously. The geometric parameters are given. (**b**) Measured spectra of the illuminating sub-pulses generated by integrating CUSP data over space at different times. Spectra are normalized to local maxima. The reference measurement by a spectrometer is plotted as the black line.

5. Characterizations of the CUSP system

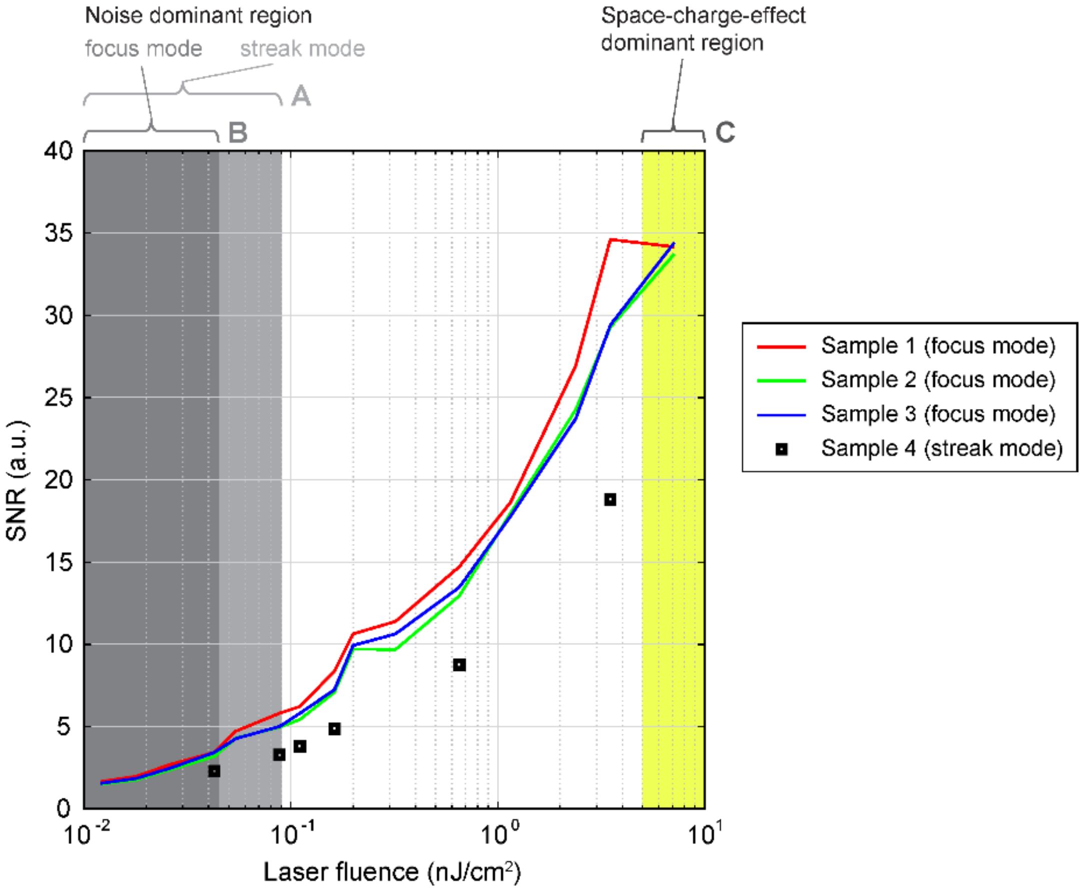

Fig. S11. Noise analysis of the CUSP imaging system. Signal-to-noise (SNR) of the raw streak camera image versus the laser fluence. Both streak mode (with an ultrafast sweeping voltage in the streak tube) and focus mode (without an ultrafast sweeping voltage in the streak tube) are measured. Three samples are imaged in focus mode. The regions where noise dominates are boxed in gray (A for streak mode and B for focus mode). The region where space-charge effect takes place is boxed in yellow (box C). 100 images were acquired for each data point for the SNR calculation.

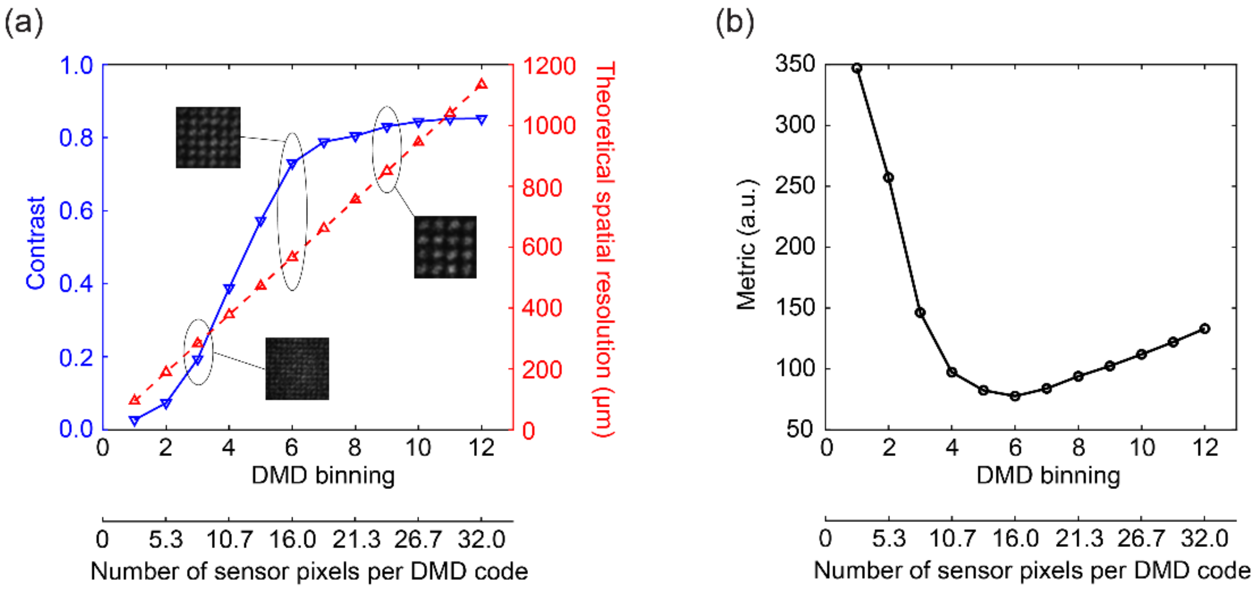

Fig. S12. Sampling rate analysis of the CUSP imaging system. Streak camera was used to acquire calibration images of periodic grids displayed by DMD. Different grid periods are determined by the DMD binning. The laser fluence used was 3 $\mathrm{nJ/cm^2}$ to give a good SNR and avoid space-charge effect at the same time. (**a**) Image contrast (left axis) and theoretical spatial resolution (right axis) versus the DMD binning size, corresponding to different numbers of sensor pixels per DMD encoding unit. (**b**) Calculated metric, defined as the theoretical spatial resolution divided by the image contrast, versus the DMD binning size.

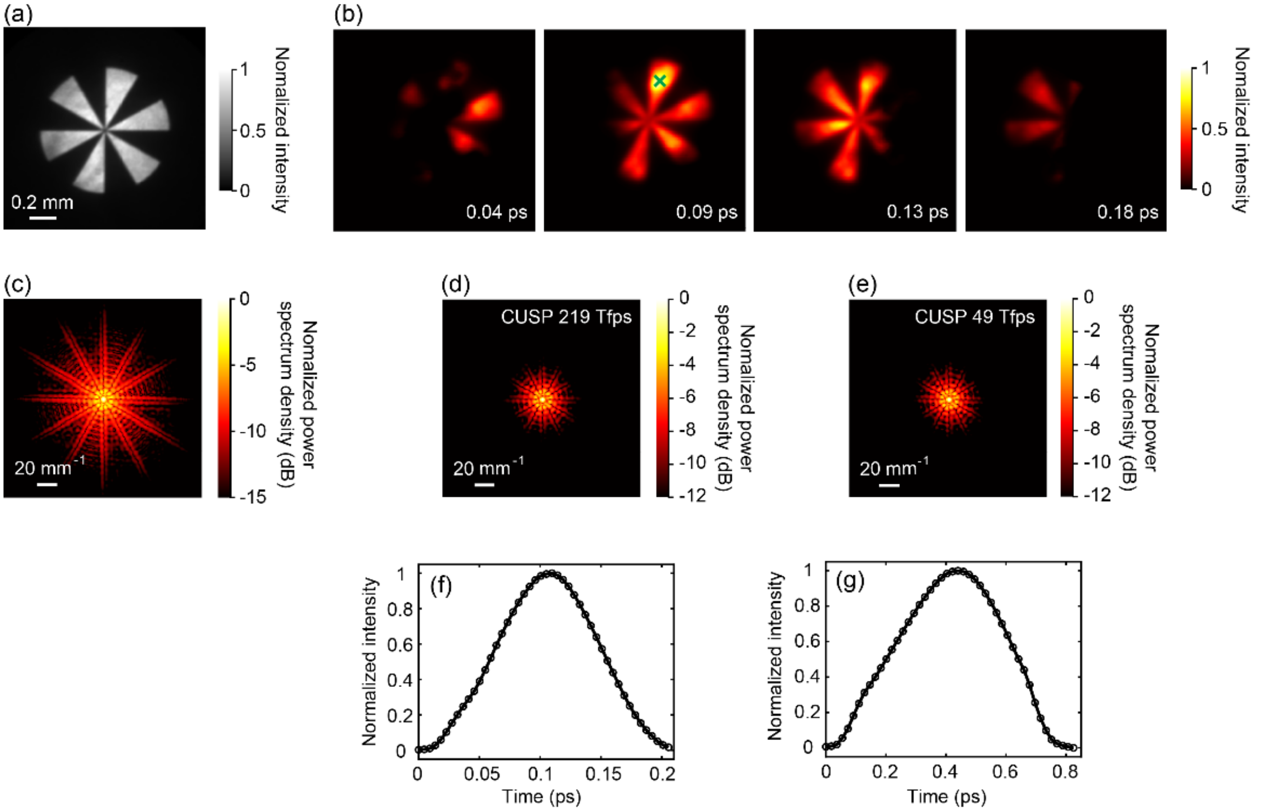

Fig. S13. Spatial and temporal resolutions of CUSP. (**a**) Static image of a spoke pattern for resolution analysis, imaged by the external camera. (**b**) Representative frames of 219-Tfps CUSP imaging of a spatiotemporally chirped pulse sweeping across the spoke pattern. (**c**) – (**e**) Spatial frequency response of (**c**) the static image in (**a**), (**d**) temporally integrated image from 219-Tfps CUSP in (**b**), and (**e**) temporally integrated image from 49-Tfps CUSP. (**f**) and (**g**) Temporal profiles of normalized intensities at a selected point from (**f**) 219-Tfps CUSP and (**g**) 49-Tfps CUSP, respectively. The point is marked as a green cross in (**b**).

Table S1: Specifications of the three example configurations of CUSP.

Specifications	Experiment 1 – Kerr	Experiment 2 – Filament	Experiment 3 – Chirped pulse
Imaging speed R (Tfps)	219	80	49
Total temporal chirp η (fs/nm)	–5.55	–41.63	111.0
Temporal chirp by stretching rod (fs/nm)	5.55	–41.63	-50.10
Length of stretching rod (mm)	10	75	90
Sequence depth N	230	640	1040
Number of sub-pulses P	5	5	5
Number of frames in one sub-pulse	46	128	208
Optimal spectrum resolution $\Delta\lambda_{\mathrm{opt}}$ (nm)	13.2	4.8	2.9
Number of resolvable spectra in one sub-pulse	2.1	5.8	9.5
Dispersion parameter μ (µm/nm)	–4.24	11.61	18.97
Grating period (lp/mm)	300	600	300
Grating and streak camera distance (mm)	13.7	17.0	61.4
Mirror tilt angle	52.0°	30.5°	38.0°
Temporal resolution Δt_{opt} (fs)	108	283	462
Sub-pulse duration Δt_{sp} (ps)	0.21	1.60	4.24
Total observation window T_{obs} (ps)	1.05	8.00	21.22
Sub-pulse separation T_{sp} (ps)	1.60	1.60	4.24
Total recording time T_{rec} (ps)	8.00	8.00	21.22
Imaging optics	4× objective and f = 100 mm tube lens	10× objective and f = 75 mm tube lens	f = 75 mm and f = 25 mm lenses
Imaging system magnification	2.22 ×	4.17 ×	0.33 ×
Relay system magnification	1 ×	1 ×	1 ×
Field-of-view (mm × mm)	1.22 × 0.23	0.51 × 0.11	6.97 × 6.97
Number of pixels in field-of-view	420 × 80	326 × 70	360 × 360
Streak camera time range (ps)	50	50	50
Streak camera gain	50	50	50
Streak camera internal magnification	1.85 ×	1.85 ×	1.85 ×

References

1 Gao, L., Liang, J., Li, C. & Wang, L. V. Single-shot compressed ultrafast photography at one hundred billion frames per second. *Nature* **516**, 74-77 (2014).

2 Liang, J., Zhu, L. & Wang, L. V. Single-shot real-time femtosecond imaging of temporal focusing. *Light: Science & Applications* **7**, 42 (2018).

3 Wang, P., Liang, J. & Wang, L. V. Single-shot ultrafast imaging attaining 70 trillion frames per second. *Nature Communications* **11**, 2091 (2020).

4 Wagadarikar, A., John, R., Willett, R. & Brady, D. Single disperser design for coded aperture snapshot spectral imaging. *Applied optics* **47**, B44-B51 (2008).

5 Llull, P. *et al.* Coded aperture compressive temporal imaging. *Optics Express* **21**, 10526-10545 (2013).

6 *Guide to Streak Cameras*, <https://www.hamamatsu.com/resources/pdf/sys/SHSS0006E_STREAK.pdf> (2008).

7 Tamamitsu, M. *et al.* Design for sequentially timed all-optical mapping photography with optimum temporal performance. *Optics Letters* **40**, 633-636 (2015).

8 Nakagawa, K. *et al.* Sequentially timed all-optical mapping photography (STAMP). *Nature Photonics* **8**, 695 (2014).

9 Liang, J. *et al.* Single-shot real-time video recording of a photonic Mach cone induced by a scattered light pulse. *Science Advances* **3**, e1601814 (2017).

10 Shen, Y. R. *The principles of nonlinear optics*. (Wiley, 2002).

Captions of Supplementary Movies

Movie S1

Ultrafast dynamics of the nonlinear Kerr effect induced by a focused ultrashort laser pulse propagating in a GGG slab, imaged by the CUSP system at 219 Tfps. This movie was acquired in the fragmented observation regime, in which five observation windows are probed by five consecutive sub-pulses. The colormap represents transmittance of the Kerr gate. The movie contains 230 frames in total.

Movie S2

Ultrafast dynamics of the filament formation and propagation inside a glass slide, pumped by a focused femtosecond laser pulse, imaged by the CUSP system at 80 Tfps. This movie was acquired at the boundary between the fragmented observation and continuous observation regimes. The colormap represents light intensity. The movie contains 640 frames in total. The images are converted to the filament's propagation coordinates in the x_p-y_p plane.

Movie S3

Ultrafast dynamics of a spatiotemporally chirped optical pulse train sweeping a printed pattern of Caltech logo, imaged by the CUSP system at 49 Tfps. This movie was acquired in the continuous observation regime. The 2D colormap uses color and grayscale to represent illumination wavelength and light intensity, respectively. The movie contains 1040 frames in total.
